# Effective Mechanical Properties of Periodic Cellular Solids with Generic Bravais Lattice Symmetry via Asymptotic Homogenization

**DOI:** 10.3390/ma16247562

**Published:** 2023-12-08

**Authors:** Padmassun Rajakareyar, Mostafa S. A. ElSayed, Hamza Abo El Ella, Edgar Matida

**Affiliations:** Department of Mechanical and Aerospace Engineering, Carleton University, Ottawa, ON K1S 5B6, Canada; padmassun.rajakareyar@carleton.ca (P.R.); hamzaella@cunet.carleton.ca (H.A.E.E.); edgar.matida@carleton.ca (E.M.)

**Keywords:** asymptotic homogenization, cartesian mesh, voxel mesh, non-orthogonal periodic basis, lattice material, Bravais lattice symmetry

## Abstract

In this paper, the scope of discrete asymptotic homogenization employing voxel (cartesian) mesh discretization is expanded to estimate high fidelity effective properties of any periodic heterogeneous media with arbitrary Bravais’s lattice symmetry, including those with non-orthogonal periodic bases. A framework was developed in Python with a proposed fast–nearest neighbour algorithm to accurately estimate the periodic boundary conditions of the discretized representative volume element of the lattice unit cell. Convergence studies are performed, and numerical errors caused by both voxel meshing and periodic boundary condition approximation processes are discussed in detail. It is found that the numerical error in periodicity approximation is cyclically dependent on the number of divisions performed during the meshing process and, thus, is minimized with a refined voxel mesh. Validation studies are performed by comparing the elastic properties of 2D hexagon lattices with orthogonal and non-orthogonal bases. The developed methodology was also applied to derive the effective properties of several lattice topologies, and variation of their anisotropic macroscopic properties with relative densities is presented as material selection charts.

## 1. Introduction

Periodic cellular solids, also known as lattice materials, are formed by tessellating a unit cell in an infinite periodicity to fill a design space. The unit cell, the representative volume element (RVE), is the smallest depiction of the lattice that has the most accurate statistical representation of its physical macroscale properties. The dimensions of the unit cell are at least an order of magnitude smaller than the characteristic length of the macroscopic structure.

While having complicated geometries that cannot be fabricated using conventional manufacturing methods, recent advances in additive manufacturing have permitted the fabrication of cellular materials with increasingly intricate structures and new topologies [1,2,3,4], opening the door for their adoption in many applications. Over the last few decades, lattice materials have been used in a wide range of applications, including acoustic and vibrational damping and reflections [5,6,7], thermal and heat exchangers [8,9,10], actuation for shape morphing structures [11,12], energy absorption [1,13,14,15], and multiscale optimization [16,17]. 

Multiscale numerical simulations to evaluate lattice material characteristics considering structural attributes at all length scales are normally challenging and computationally expensive. Thus, homogenization methods are developed to analyze the effective qualities of a multiscale material such that its macroscale properties may be represented statistically. 

A number of analytical, numerical, and experimental studies have been proposed in the literature [18,19,20,21,22,23,24,25,26] to characterize the effective properties of lattice materials. Some of the proposed homogenization techniques include energy methods [18,19,20,21], the wavelet-reduced order model [22], the Cauchy–Born hypothesis [23,24,25,26], discrete homogenization [27,28,29], and asymptotic homogenization [30,31,32,33,34]. 

Some notable works [35,36,37] have provided closed-form representations of the effective mechanical characteristics of lattice materials. Their methods, however, were only applicable to simple topology with straightforward arrangement of lattice cells.

The characteristics of planar lattice materials have also been homogenized using matrix-based methods based on Bloch’s theorem and the Cauchy–Born hypothesis [24,25]. In order to derive the material’s macroscopic stiffness parameters, Hutchinson and Fleck [24] first converted the microscopic nodal deformations of a lattice in terms of the macroscopic strain field. A method was developed to describe cell topologies, such as the Kagome lattice and the Triangular–Triangular lattice, which have a particular degree of symmetry. This approach was expanded by ElSayed and Pasini [25] and ElSayed [38] to handle planar topologies that can have any arbitrary cell geometry. A more generic matrix-based method for the analysis of arbitrary bi-dimensional and tri-dimensional cell topologies with open and closed cells was described by Vigliotti and Pasini [23,26,39].

The characterization of cellular materials has also been effectively accomplished using discrete homogenization approaches [27,28,29]. These methods simulate the lattice cell walls using discrete elements like beam or rod elements. The homogenized characteristics are produced by converting the discrete sum of equilibrium equations into a continuous relation of stress and strain.

The asymptotic homogenization (AH) theory has been effectively used among other numerical methods for predicting the effective mechanical characteristics of periodic lattice materials [30,31,32,33,34,40,41,42,43,44]. Their results have been validated by experimental tests, demonstrating the effectiveness of the AH method [45,46,47,48]. The main discrepancy between the experimental and the theoretical analyses was mainly due to defects and deviations present in the manufactured part. The defects invalidated the periodic boundary condition and caused deviations in the measured experimental values. A standout benefit of AH in comparison to other homogenization systems is its ability to precisely identify the stress distribution in the lattice unit cell, which can then be utilized for an in-depth analysis of the strength and damage of heterogeneous periodic materials [29,49].

The double-scale AH method, considering the use of 2D iso-parametric plate elements, was employed by Andreassen and Andreasen [33] to determine the elastic properties, thermal expansion, thermal conductivity, and fluid permeability of 2D periodic cellular lattices and composite materials. Andreassen and Andreasen [33] were able to analyze RVE’s cell envelope with monoclinic, orthorhombic, tetragonal, and hexagonal periodicities by changing the shape of the 2D iso-parametric quadrilateral plate element.

Considering the use of solid elements for discrete homogenization techniques, the homogenized elastic tensor could be obtained by applying unit strains and performing volume averaging of the stresses and strains in the discretized elements. This approach has been performed using popular commercial finite element packages, such as Abaqus [50] and ANSYS Material Designer [51]. The main limitation for these commercial applications adopting this approach is that they are only developed for RVEs with orthorhombic, tetragonal, or cubic periodicity [50,51,52] mainly due to the limitations imposed by the meshing process, leading to an incompatibility while applying the periodic boundary conditions. When discretizing the RVE, the locations and the number of nodes along a periodic boundary face would not necessarily match while applying periodic boundary conditions.

**Figure 1:**
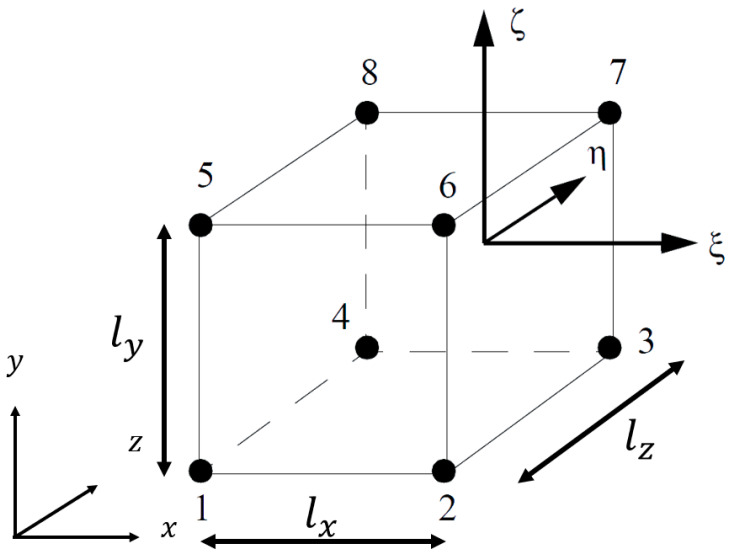
Iso-parametric hexahedral voxel element, with the global coordinate system (x,y,z), local coordinate system (ξ,ζ,η), and voxel edges aligned with a global cartesian coordinate system with lengths ($l_{x},l_{y},l_{z}$), respectively.

Dong et al. [34] expanded the work of Andreassen and Andreasen [33] by considering the use of 3D solid elements (iso-parametric hexahedral element shown in Figure 1, also hereafter referred to as 3D voxel). The code developed by Dong et al. [34] was limited to analyzing the periodic cell envelope with the orthogonal periodic basis.

This paper expands on the work performed by Dong et al. [34]. The prior work only considered cell envelopes with orthogonal periodicity, whereas the current work expands upon this by (a) proposing methods to discretize unit cells with non-orthogonal bases and (b) applying approximated periodic boundary conditions for non-orthogonal cell envelopes to evaluate the homogenized elastic properties. 

The paper is organized into five sections. After the introduction, in Section 2, the methodology for discretizing the geometry and approximating the periodic boundary condition, along with the numerical homogenization process for determining high fidelity homogenized elastic properties, are presented. In Section 3, the validation for the numerical analysis is detailed. The developed methodology is then applied to triclinic and monoclinic Bravais grid lattice topologies, and the results are documented in Section 4. The paper is concluded in Section 5. 

The developed methodology was also used to derive the effective properties of 35 lattice topologies, and variation of their anisotropic macroscopic properties with relative densities which is presented in the appendices included in the Supplementary File.

## 2. Methodology

The periodic cellular solids can have an open or a closed cell construction. The former can be modelled as a micro-truss-like structure, while the latter is commonly represented with shells and plates. In this paper, an open-cell micro-truss with circular cross-section elements is considered. 

**Figure 2:**
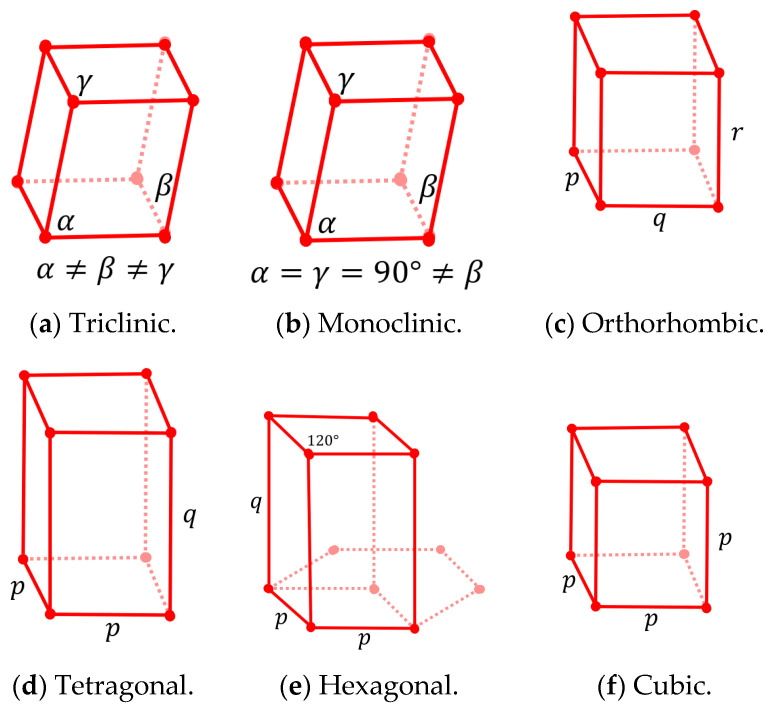
Primitive Bravais lattices. α, β, and γ are angles (α≠β≠γ), whereas p, q and r are lengths (p≠q≠r).

The numerical homogenization performed in this paper is based on the asymptotic double-scale homogenization [30,31,32,33,53,54] with a workflow written in Python [55]. Here, the periodic cellular lattices are categorized, based on the shape of the cell envelope, as either orthogonal or non-orthogonal. Cell envelopes with orthogonal periodic bases could be part of an orthorhombic, tetragonal, or cubic Bravais lattice system, as shown in Figure 2. On the other hand, non-orthogonal cell envelopes have non-orthogonal periodic bases and could be part of triclinic, monoclinic, and hexagonal systems.

### 2.1. Representative Volume Element

**Figure 3:**
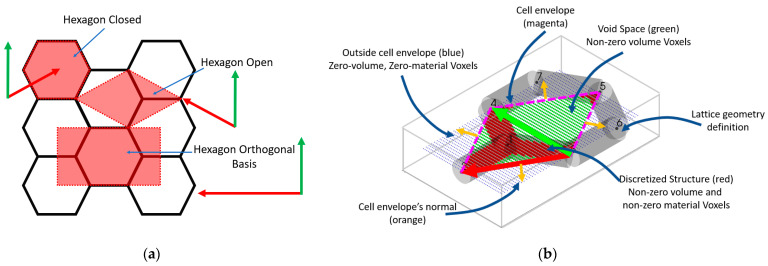
(**a**) Honeycomb lattice RVE with multiple cell envelope definitions. Periodic bases are shown with red and green arrows for the three proposed envelopes. (**b**) Visualization of the 2D Open Hexagon RVE, RVE’s envelope, voxels, and periodic basis. Voxels’ shape is not to scale.

**Figure 4:**
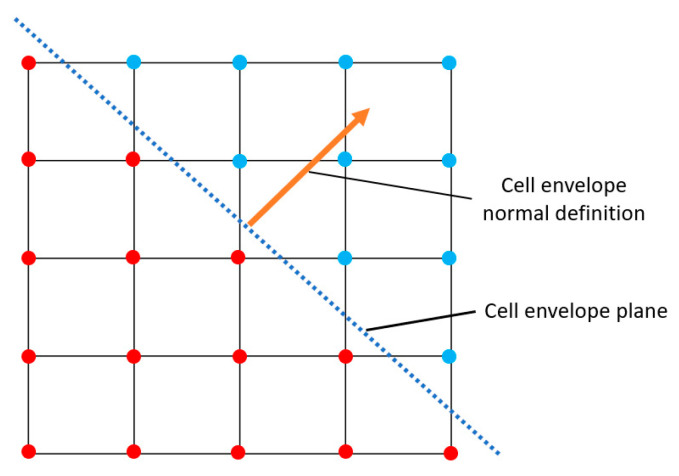
Cell envelope definition for filtering voxels with zero and non-zero volume. Red voxel centers are considered to be inside the cell envelope, whereas blue-coloured voxel centers are outside the cell envelope based on the normal direction of the cell envelope (orange arrow).

The RVE of a lattice is its unit cell represented by a cell envelope and the lattice structure, as shown in Figure 3. This figure shows the honeycomb lattice topology with different possibilities of cell envelope representation, including both orthogonal and non-orthogonal bases. Figure 3b shows a discretized 2D hexagon geometry with a non-orthogonal cell envelope. Three different types of voxels are shown in this figure. The green voxels represent the voids within the RVE’s cell envelope, and they have a non-zero volume and zero material property. The green voxels are only considered when calculating total volume but ignored when finding periodic node pairs and calculating the stiffness matrix. The red voxels represent the discretized geometry, with both non-zero material property and non-zero volume. The blue voxels are assigned a zero volume and zero material property because they are outside the RVE’s cell envelope. The cell envelope is defined as a face with a normal pointing outside the cell envelope, as shown in Figure 4. Based on the cell envelope’s plane definition, any voxel elements that are outside the cell envelope (same side as the normal vector) are removed during the analysis.

The parent material property for the individual voxel is defined using the two Lame’s parameters, namely, λ and μ, which are expressed as:

(1) $$\lambda =\frac{vE}{\left(1+v\right)\left(1-2v\right)}\;\mu =\frac{E}{2(1+v)}$$

where E and ν are the Young’s modulus and the Poisson’s ratio, respectively. The analysis presented in this paper employs isotropic steel with $E_{iso}={2.0\times 10}^{11}$ Pa and $\nu_{iso}=0.3$, which correspond to lame parameters of $\lambda =1.153\times 10^{11}$ Pa and $\mu =7.692\times 10^{11}$ Pa.

The voxel element is formulated as an iso-parametric brick element whose edges are orthogonal with lengths of $l_{x}$, $l_{y}$ and $l_{z}$ aligned in the global cartesian coordinate system. The individual voxels with non-zero volume, which are inside the cell envelope, are treated as having an isotropic material property, and the element’s stiffness material matrix ($C^{\left(e\right)}$) is formulated as:

(2) $$C^{\left(e\right)}=\lambda^{\left(e\right)}\left[\begin{array}{cccccc} 1 & 1 & 1 & 0 & 0 & 0\\ 1 & 1 & 1 & 0 & 0 & 0\\ 1 & 1 & 1 & 0 & 0 & 0\\ 0 & 0 & 0 & 0 & 0 & 0\\ 0 & 0 & 0 & 0 & 0 & 0\\ 0 & 0 & 0 & 0 & 0 & 0 \end{array}\right]+\mu^{\left(e\right)}\left[\begin{array}{cccccc} 2 & 0 & 0 & 0 & 0 & 0\\ 0 & 2 & 0 & 0 & 0 & 0\\ 0 & 0 & 2 & 0 & 0 & 0\\ 0 & 0 & 0 & 1 & 0 & 0\\ 0 & 0 & 0 & 0 & 1 & 0\\ 0 & 0 & 0 & 0 & 0 & 1 \end{array}\right]$$

### 2.2. Discretization and Approximation of the Periodic Boundary Condition

The process to determine the translational periodicity of the node pairs is to translate the coordinates of a node using the periodic basis vector and then find the closest node within a prescribed search radius. 

A periodic physical quantity of a structure can be represented as:

(3) $$F\left(x+NY\right)=F\left(x\right)$$

where $x={\left[x_{1},x_{2},x_{3}\right]}^{T}$ is the position vector of a point where the physical quantity F is evaluated, $N=\lceil n_{1},n_{2},n_{3}\rfloor$ is a 3×3 diagonal matrix which consists of arbitrary integer values, and $Y={\left[Y_{1},\;Y_{2},\;Y_{3}\right]}^{T}$ is a vector that denotes the period of the structure, where this value could be a scalar, vector, or tensor function of x [30]. 

The static response of a periodic structure can then be represented as:

(4) $$\sigma_{ij}=C_{ijkl}(x)\varepsilon_{kl}=C_{ijkl}(x+NY)\varepsilon_{kl}\;i,j,k,l\in \{1,2,3\}$$

where $C_{ijkl}$ is the stiffness tensor, $\varepsilon_{kl}$ is the strain tensor, and $\sigma_{ij}$ is the stress tensor. By definition of x,N, and Y, the stiffness tensor $C_{ijkl}$ can be expanded as

(5) $$C_{ijkl}\left(x_{1}+n_{1}Y_{1},x_{2}+n_{2}Y_{2},x_{3}+n_{3}Y_{3}\right)=C_{ijkl}\left(x_{1},x_{2},x_{3}\right)\;i,j,k,l\;\in \{1,2,3\}$$

The voxel nodes have been discretized such that the nodal coordinates ($x_{1}^{i},\;x_{2}^{i},\;x_{3}^{i}$) of the ith node are defined as:

(6) $$\left(\begin{array}{c} x_{1}^{i}\\ x_{2}^{i}\\ x_{3}^{i} \end{array}\right)=\left(\begin{array}{c} A_{1}l_{x}+O_{x}\\ A_{2}l_{y}+O_{y}\\ A_{3}l_{z}+O_{z} \end{array}\right),\;where\;\begin{array}{c} A_{1}=\left\{0,1,\;2,\;\ldots ,\;x_{divs}\right\}\\ A_{2}=\left\{0,\;1,\;2,\;\ldots ,\;y_{divs}\right\}\\ A_{3}=\left\{0,\;1,\;2,\;\ldots ,\;z_{divs}\right\} \end{array},\;i=\{1,\;2,\;\ldots ,\;\left(x_{divs}+1\right)*\left(y_{divs}+1\right)*\left(z_{divs}+1\right)\}$$

where, $A_{1},A_{2},$ and $A_{3}$ are integers and {$O_{x},\;O_{y},\;O_{z}$} is the origin. The i superscript indicates node numbers; $l_{x}$, $l_{y}$, and $l_{z}$ are the lengths of the voxel along the global x, y, and z axis, respectively; and the $x_{divs}$, $y_{divs}$ and $z_{divs}$ are integers denoting the number of voxel discretization along the global x, y, and z axes, respectively. Typically, $l_{x}$, $l_{y}$, and $l_{z}$ are the same for all the voxels and can be calculated as:

(7) $$\left(\begin{array}{c} l_{x}\\ l_{y}\\ l_{z} \end{array}\right)=\left(\begin{array}{c} \frac{\max\left(x_{1}^{i}\right)-min(x_{1}^{i})}{x_{divs}}\\ \frac{\max\left(x_{2}^{i}\right)-min(x_{2}^{i})}{y_{divs}}\\ \frac{\max\left(x_{3}^{i}\right)-min(x_{3}^{i})}{z_{divs}} \end{array}\right)\;and\;\left(\begin{array}{c} O_{x}\\ O_{y}\\ O_{z} \end{array}\right)=\left(\begin{array}{c} min(x_{1}^{i})\\ min(x_{2}^{i})\\ min(x_{3}^{i}) \end{array}\right)$$

The periodicity vector Y can be decomposed such that:

(8) $$Y=\left(\begin{array}{c} Y_{1}\\ Y_{2}\\ Y_{3} \end{array}\right)=\left(\begin{array}{c} B_{1}l_{x}\\ B_{2}l_{y}\\ B_{3}l_{z} \end{array}\right)+\left(\begin{array}{c} G_{1}\\ G_{2}\\ G_{3} \end{array}\right):\;\begin{array}{c} B_{1},\;B_{2},\;B_{3}\in Z\\ \begin{array}{c} G_{1},\;G_{2},\;G_{3}\in R\\ G_{1}<l_{x},\;G_{2}<l_{y},\;G_{3}<l_{z}, \end{array} \end{array}\;$$

where $B_{1},B_{2},$ and $B_{3}$ are integers and $G_{1},G_{2},$ and $G_{3}$ are the residual or the remainder which are smaller than the lengths of the voxel $l_{x},l_{y},$ and $l_{z}$, respectively. 

For each node of the voxel defined by Equation (6) that is part of the RVE’s cell envelope, there exists a periodic node pair that is also part of the discretized RVE’s cell envelope.

**Figure 5:**
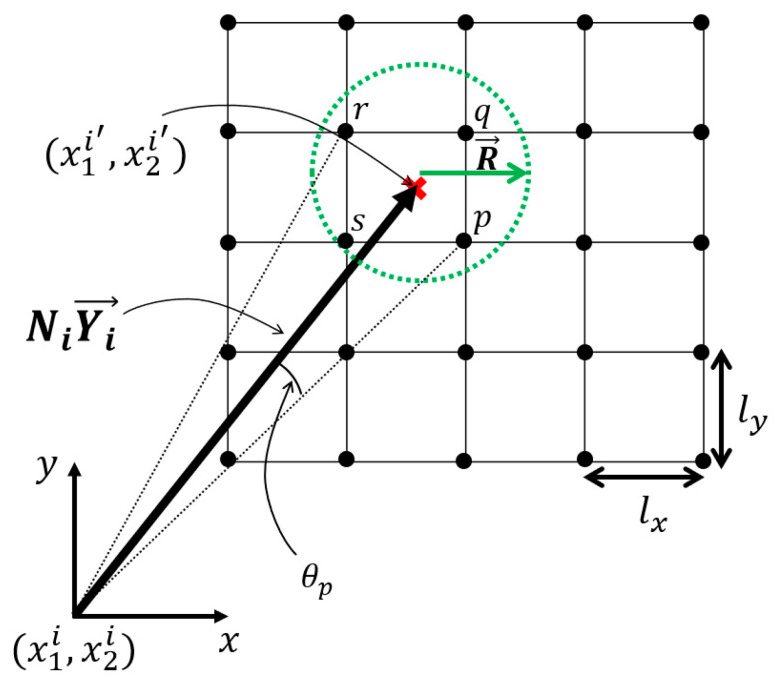
Determination of a periodic node pair for node i located at ($x_{1}^{i}$, $x_{2}^{i}$) with its periodic pair i′ at $N_{i}\vec{Y_{i}}$ and the search radius $\vec{R}$ (green arrow) required to search the approximate periodic node pair due to the difference between the voxel element basis and RVE’s periodic basis. This search radius can be used with a KD-Tree algorithm [56] to query the closest point.

If any of the residuals $G_{1}$, $G_{2}$, or $G_{3}$ are non-zero, then it would not be possible to find the exact node that is part of the voxels, as shown in Figure 5, (which leads to approximating the periodic node pairs, causing some numerical errors which are discussed in Section 3.1 and Section 3.4). Approximating the location of the periodic node pair involves ignoring the residuals ($G_{i}$). A perfect voxel mesh can be obtained by minimizing the residual term. For an orthogonal periodic basis, aligned with the global cartesian coordinate system, the residuals are automatically zero. But for an RVE with a non-orthogonal periodic basis, the $x_{divs}$, $y_{divs}$, and $z_{divs}$ must be altered to minimize the residuals.

Thus, to find the periodic node pair for an RVE with a non-orthogonal periodic basis, a local search must be conducted within a radius of R, such that at least one point lies within the defined search radius, as shown in Figure 5. If the periodic node is not found for the node in the cell boundary, or the node pair is incorrectly identified, it can introduce small numerical errors. A suggested search radius is proposed as follows:

(9) $$R=\max\left(\begin{array}{c} 0.51\;max\left(l_{x},l_{y},l_{z}\right)\\ 0.51\left(\sqrt{l_{x}^{2}+l_{y}^{2}+l_{z}^{2}}\right) \end{array}\right)$$

For a coarse mesh (where $l_{x},l_{y},l_{z}$ values are similar or larger relative to the truss radius), it is suggested to use angular constraints, where the angle $\theta_{p}$, shown in Figure 5, is more than 5°, rather than the suggested radius constraint in Equation (9). The angle $\theta_{p}$, as shown in Figure 5, can be calculated by performing the dot product operation between the two normalized vectors, $\vec{Y}$ and $\vec{x^{i}p}$. The misalignment of the periodic basis with the voxel’s element natural coordinate system introduces slight numerical errors, which are discussed in Section 3.

To determine the periodicity of the lattice, the nodal coordinates of all the voxels are used to create a k-d tree from [56] to implement a fast–nearest neighbour algorithm. Then, similar to Equation (5), an offset from Equation (8) is applied to all of the voxel coordinates based on all of the unique combinations of the basis, and the closest point within a certain minimum distance R and a minimum angle θ is chosen as its periodic nodal pair. Unit cells with the orthogonal periodic basis that align with the voxel’s natural coordinate system do not need the minimum distance and the angle restrictions. But for a lattice geometry with a periodic basis that is not an integer multiple of the voxel’s natural coordinate system, the process of finding the periodic nodal pair requires an additional step because the offset coordinate is not coincident with the second nodal pair. Thus, a local search must be conducted to find the closest point that is subject to minimum distance and angle restrictions described by (9).

### 2.3. Numerical Homogenization

The asymptotic homogenization process is based on the double-scale expansion theory [30,31,32,33,53,54]. The homogenized macroscopic elasticity tensor can be written as:

(10) $$E_{ijkl}^{H}=\frac{1}{\left|V\right|}\int_{V}E_{pqrs}\left(\varepsilon_{pq}^{0\left(ij\right)}-\varepsilon_{pq}^{\left(ij\right)}\right)\left(\varepsilon_{rs}^{0\left(kl\right)}-\varepsilon_{rs}^{\left(kl\right)}\right)dV,\;\;i,j,k,l,p,q,r,s\;\in \{1,2,3\}$$

where V is the volume of the base RVE, $E_{pqrs}$ is the locally varying stiffness tensor, $\varepsilon_{pq}^{0\left(ij\right)}$ represents the macroscopic strain, and $\varepsilon_{pq}^{\left(ij\right)}$ is the microscopic strain (locally periodic). The superscript H denotes the homogenized quantity. More details for 3D and 2D voxels can be found at [33,34]. $\varepsilon_{pq}^{\left(ij\right)}$ is defined as:

(11) $$\varepsilon_{pq}^{\left(ij\right)}=\varepsilon_{pq}\left(\chi^{ij}\right)=\frac{1}{2}\left(\chi_{p,q}^{ij}+\chi_{q,p}^{ij}\right),\;p,q\;\in \{1,2,3\}$$

Based on displacement fields $\chi^{\;kl}$, which are found by solving the elasticity equations with prescribed macroscopic strains:

(12) $$\int_{V}^{}E_{ijpq}\varepsilon_{ij}\left(\nu \;\right)\varepsilon_{pq}\left(\chi^{kl}\right)dV=\int_{V}^{}E_{ijpq}\varepsilon_{ij}\left(\nu \;\right)\varepsilon_{pq}^{0\left(kl\right)}dV\;i,j,k,l,p,q\in \{1,2,3\}$$

where ν is the virtual displacement field. Homogenization is normally performed numerically using discretization and finite element method to solve Equation (12).

## 3. Validation

In this section, the analysis of a voxelized RVE with a non-orthogonal base is validated against results of a commercial finite element software for the same lattice topology but represented by a different RVE with orthogonal bases, as reported by Gibson and Ashby [36], Vigliotti and Pasini [26], and other homogenization codes by Andreassen and Andreasen [33], as well as Dong et al. [34]. For low relative density lattice, the results of the voxelized non-orthogonal hexagon are compared against similar non-orthogonal hexagons but formulated using beam theory [27,28,29]. Grid convergence studies have been performed in this section, and the numerical errors caused by both the voxel meshing and the periodic boundary condition approximation processes are discussed in detail. The material used in the validation study is isotropic steel with $E_{iso}={2.0\times 10}^{11}$ Pa and $\nu_{iso}=0.3$.

### 3.1. Grid Convergence Study

**Figure 6:**
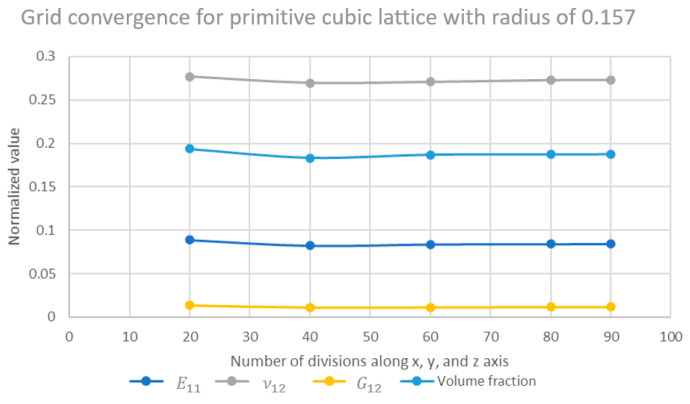
Grid convergence study.

For the grid convergence study, the lattice materials’ truss radius is held constant, and the number of divisions along the global axis is varied. For the first mesh sensitivity study, a 3D primitive cubic lattice with an orthogonal cell envelope was analyzed, and the results are shown in Figure 6. The primitive cubic lattice, which had a unit length of 1 with a truss radius of 0.157, was targeted, which converged to a volume fraction of 0.18. The convergence of elastic properties was satisfied around a discretization of 60 voxels in each direction. 

**Figure 7:**
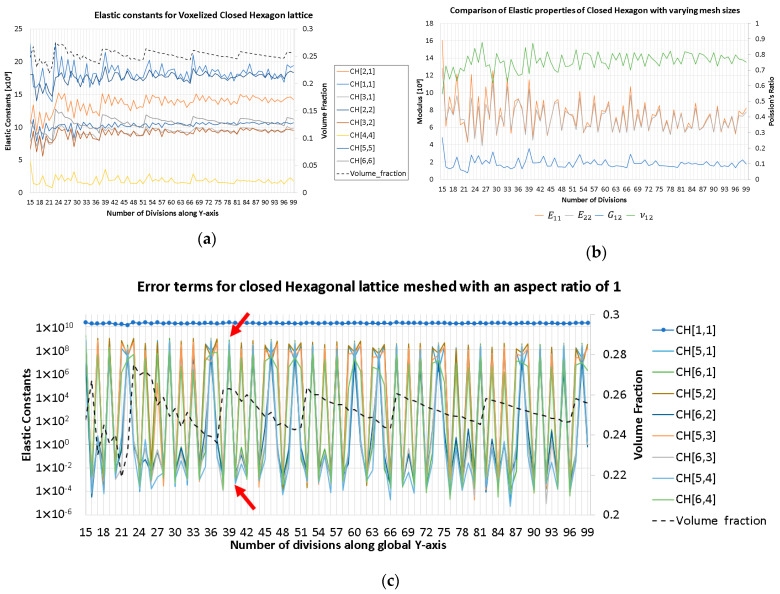

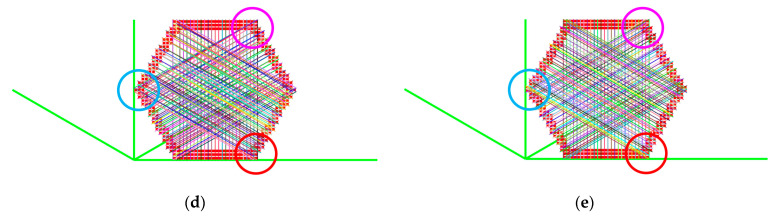
Numerical error analysis of a closed hexagon with voxel aspect ratios of unity. (**a**) The large non-zero elastic constants. For a constant truss radius, the volume fraction changes due to the voxel meshing process, and the volume fraction converges for a smaller voxel mesh. (**b**) The homogenized effective properties of the hexagon lattice, where the fluctuations are caused by the voxel meshing process and approximating the periodic boundary conditions. (**c**) The residual terms in a log plot, where the residuals fluctuate between a high and a low value, corresponds to the error caused by approximating the periodic boundary conditions. (compare colored circles in (**d**,**e**)) The periodic boundary conditions for the voxelized hexagon lattice are marked in (**c**). (**d**) Large error (AR ≈ 1) for 46 by 39 divisions, VF = 0.263. This voxelized hexagon corresponds to the upper red arrow shown in (**c**). (**e**) Small error (AR ≈ 1) for 47 by 40 divisions, VF = 0.262. This voxelized hexagon corresponds to the lower red arrow shown in (**c**).

**Figure 8:**
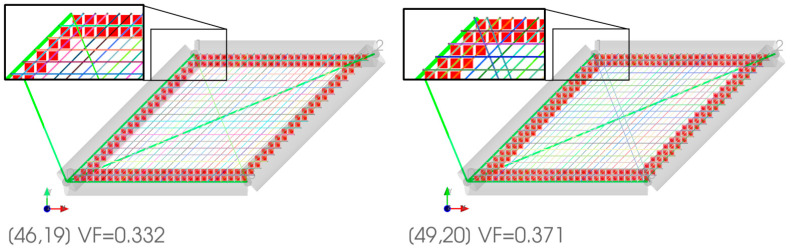
Variation of volume fraction due to increasing the number of voxel divisions in the y-direction while maintaining an aspect ratio of unity. The voxel’s center coordinate has been represented as smaller voxels. The periodic basis is plotted as a bold green line.

Then, a 2D closed hexagon lattice was analyzed, and a volume fraction of 0.25 was targeted using a radius of 0.117 units. The length of the truss was 1 unit, and the cell angle was 60°. The elastic constants of the stiffness matrix and the elastic properties are presented in Figure 7 It was observed that increasing the number of divisions affects the volume fraction of the discretized geometry; this is due to the voxel meshing process, where at some critical point, the volume fraction increases due to the voxel center coordinate suddenly being included as part of the truss radius (refer to Figure 8). In the plots below, the other quantities generally follow a similar trend as the volume fraction (black dashed lines). However, the observed discrepancy is generally due to the errors in approximating periodicity, which generally converges while refining the mesh.

The theoretical zero terms presented in an orthotropic stiffness matrix are plotted in Figure 7 above. It can be observed that the supposed zero terms fluctuate between a high and low value. The higher values are at least two orders of magnitude smaller than the main diagonals of the matrix, whereas the lower error terms are at least eight orders of magnitude smaller than the main diagonals. The main cause for this is because the periodicity is discretized where the remainder term ($G_{i}$) mentioned in Equation (8) is minimized; this means that the periodicity basis vector could be resolved as an integer multiple of the voxel lengths.

**Figure 9:**
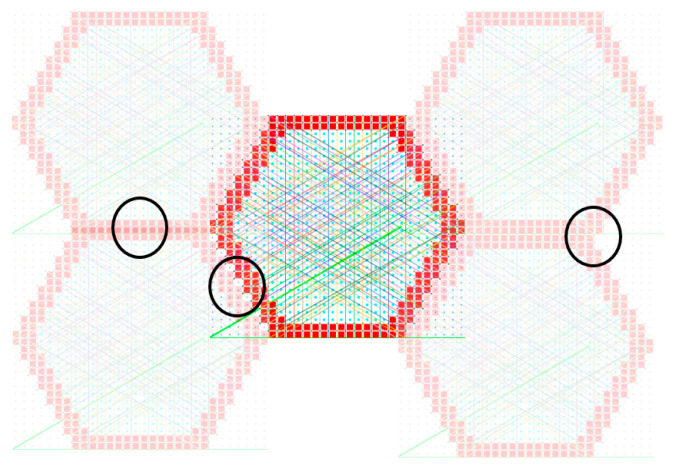
Visualization of mismatched periodicity (black circles) and its effect on lattice symmetry. The periodic node pairs are connected using randomly colored lines.

The effect of reduced remainder term leads to a case where the periodic nodes are matched in an orderly fashion, as shown in Figure 8 and Figure 7b. When comparing the periodicity lines from Figure 7a’s blue circle to the magenta circle, the periodicity lines are not parallel, as shown in Figure 7b. Furthermore, comparing the periodicity from the blue circle to the red circle, a single periodic node is shared with two other nodes; this can lead to the periodicity being mismatched. Several of these scenarios are visualized in Figure 9. Based on the visualization, the planes of symmetry are disrupted when periodicity is not matched properly, which causes the orthotropic cellular lattices to have smaller magnitude non-zero terms like a monoclinic or a triclinic material. An in-depth analysis is presented in Section 3.4.

### 3.2. Comparison with ANSYS

**Figure 10:**
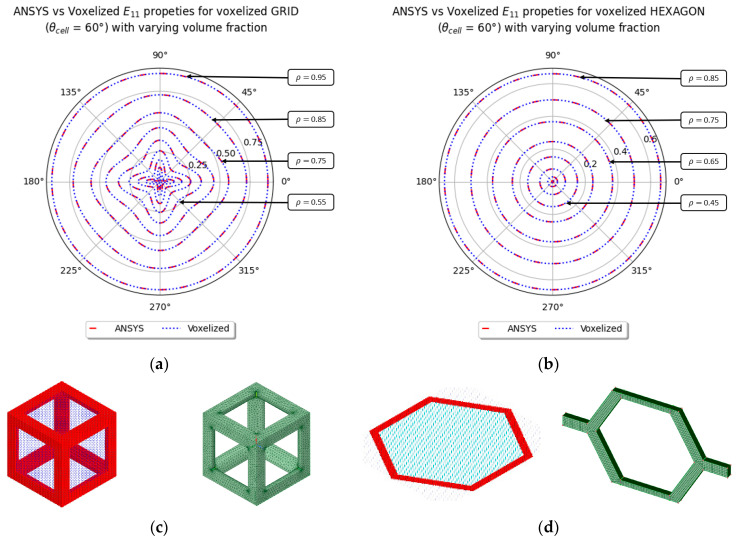
Comparison of normalized $E_{11}$ anisotropy plot at multiple volume fractions for (**a**) voxelized grid with ANSYS’ cubic lattice model, and (**b**) voxelized hexagon with non-orthogonal periodic basis and ANSYS’ Honeycomb lattice cell with orthogonal periodic basis. (**c**) Voxelized grid and ANSYS’ cubic lattice model. (**d**) Voxelized hexagon with non-orthogonal periodic basis and ANSYS’ Honeycomb model with orthogonal periodic basis.

The results obtained from the voxelization process are compared with the results from ANSYS Material Designer, which can perform homogenization of elastic and thermal properties for RVE with an orthogonal periodic basis. The results obtained from ANSYS are comparable with the results obtained with the voxelized method, with differences of less than 2%. The anisotropy plot for the primitive cubic lattice and hexagon lattice is shown in Figure 10. The primitive cubic lattice in ANSYS was meshed using tetrahedrons, and the meshing strategy made sure that the meshes in all of the faces were similar, such that the periodicity of the lattice could be found easily. The hexagon lattice in ANSYS follows a similar meshing strategy, but the Honeycomb was meshed with a mix of hexahedral (brick) and tetrahedral elements. However, the cell envelope’s faces are meshed using brick elements, which ensures that the cell envelope’s nodes can be matched easily to apply the periodic boundary conditions. ANSYS also provides an option to calculate the homogenized properties by applying symmetric boundary conditions for some of the simpler lattices.

Three planes of symmetry exist in the x, y, and z-planes for both the primitive cubic lattice in 3D and the hexagon lattice in 2D (with periodic conditions imposed in the out-of-plane). Thus, the homogenized elastic properties for these two lattices can be considered as an orthotropic. Theoretically, the homogenized elasticity tensor in the Voigt notation for an orthotropic lattice cell takes the form of:

(13) $$\left[C_{ij}^{H}\right]=\left[\begin{array}{cccccc} C_{11} & C_{12} & C_{13} & 0 & 0 & 0\\ C_{21} & C_{22} & C_{23} & 0 & 0 & 0\\ C_{31} & C_{32} & C_{33} & 0 & 0 & 0\\ 0 & 0 & 0 & C_{44} & 0 & 0\\ 0 & 0 & 0 & 0 & C_{55} & 0\\ 0 & 0 & 0 & 0 & 0 & C_{66} \end{array}\right]\;i,j\;\in \{1,2,3\}$$

**Figure 11:**
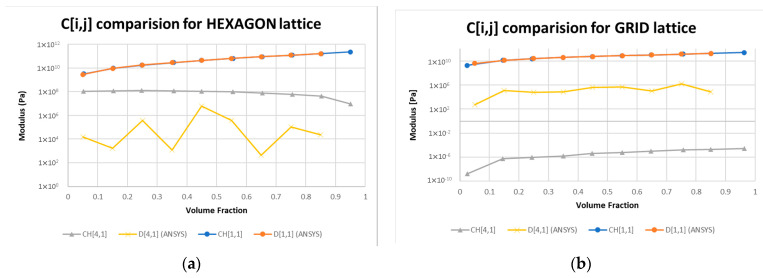
Comparison of the [4,1] term of the homogenized elastic tensor for (**a**) hexagon lattice and (**b**) grid lattice with the [1,1] term for a material of $E_{iso}$ = 2 $\times \;10^{11}$ and $nu_{iso}$ = 0.3. The CH and D labels correspond to the data from the voxelization and ANSYS material modeller, respectively.

Due to some small numerical residuals, the zero terms shown in Equation (13) were not zeros when the hexagon and the cubic lattice were homogenized. A comparison of the terms for $C_{41}^{H}$ for the hexagon and cubic lattice is shown in Figure 11. The data exported from ANSYS, which is marked as “D [1,1]” and “D [4,1]” in the figures, are from the grid and the hexagon lattice cell, respectively.

Based on the data presented in Figure 11, it can be observed that results from ANSYS have a lower $C_{41}^{H}$ term for the hexagon lattice, and a higher error for the grid lattice, in comparison to the voxelized homogenization process developed in this paper. This small numerical error is caused by the meshing scheme, as the grid lattice was meshed using tetrahedrons, while the hexagons were meshed mostly using brick elements, as shown in Figure 10c,d, respectively. In the next section, numerical errors caused by approximating periodicity are discussed.

### 3.3. RVE Rotation

The anisotropic behaviour of the elastic properties of the lattice cell is investigated by rotating the microstructure of the unit cell with respect to the applied macroscopic strain field; this is carried out by applying the rotation tensor as follows:

(14) $${E_{ijkl}^{H}}^{\prime }=Q_{ip}Q_{jq}Q_{kr}Q_{ls}E_{pqrs}^{H}\;i,j,k,l,p,q,r,s\;\in \{1,2,3\}$$

where Q is the orthogonal tensor, which corresponds to an orthogonal transformation from $x_{i}^{\prime }$ to $x_{i}$ basis. The $C_{ij}^{H}$ in the Voigt notation is expanded to $E_{ijkl}^{H}$, and an Einstein summation [57] was performed, where $Q_{ip}=Q_{jq}=Q_{kr}=Q_{ls}=Q$.

The 2D anisotropy plot produced in this paper (shown in Figure 10) corresponds to a rotation in the $x_{3}$ the axis of the global coordinate system, where:

(15) $$Q_{ip}=\left[\begin{array}{ccc} cos(\alpha ) & -sin(\alpha ) & 0\\ sin(\alpha ) & cos(\alpha ) & 0\\ 0 & 0 & 1 \end{array}\right],\;0\leq \alpha \leq 2\pi$$

The 3D anisotropy plot produced in this paper corresponds to a rotation in the $x_{3}$ axis and the $x_{2}$ axis, where:

(16) $$Q_{ip}=\left[\begin{array}{ccc} cos(\alpha ) & -sin(\alpha ) & 0\\ sin(\alpha ) & cos(\alpha ) & 0\\ 0 & 0 & 1 \end{array}\right]\left[\begin{array}{ccc} cos(\beta ) & 0 & sin(\beta )\\ 0 & 1 & 0\\ -sin(\beta ) & 0 & cos(\beta ) \end{array}\right],\;\begin{array}{c} 0\leq \alpha \leq 2\pi \\ \;0\leq \beta \leq 2\pi \end{array}$$

**Figure 12:**
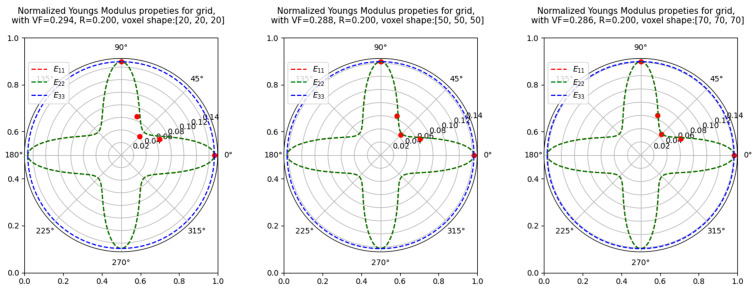
Anisotropy plot of the Youngs Modulus Elastic property of a 3D primitive cubic lattice cell, where the red circles mark the $E_{11}$ values at multiple rotation cell angles similar to the rotated cells shown in Figure 13. The dashed $E_{11}$ values are overlapped with the $E_{22}$ values due to geometrical symmetry.

Using the compliance matrix by finding the inverse of the elasticity matrix, the following elastic properties can be determined:

(17) $$S_{ij}=E_{ij}^{-1}\;E_{x}^{H}=\frac{1}{S_{11}},\;E_{y}^{H}=\frac{1}{S_{22}},\;E_{z}^{H}=\frac{1}{S_{33}},\;G_{xy}^{H}=\frac{1}{S_{44}},\;G_{yz}^{H}=\frac{1}{S_{55}},\;G_{xz}^{H}=\frac{1}{S_{66}},\;\nu_{xy}^{H}=-\frac{S_{12}}{S_{11}},\;\nu_{xz}^{H}=-\frac{S_{13}}{S_{11}},\;\nu_{yz}^{H}=-\frac{S_{23}}{S_{22}}$$

where $S_{ij}$ is the compliance matrix, and $E_{ij}$ is the elastic stiffness matrix. The sensitivity of the primitive cubic lattice’s elastic property to the cell rotation was performed and presented in Figure 12. The radius for each plot was adjusted such that a volume fraction of 0.3 could be achieved. The RVE was rotated along the z-axis by an angle of α, and the $E_{11}$ was plotted in the anisotropy plot, which was plotted for α=0°. For a volume fraction of 0.3, it was noted that 50 voxel discretization was sufficient to prove that applying the rotation matrix to the homogenized elastic tensor is equivalent to rotating the RVE.

**Figure 13:**
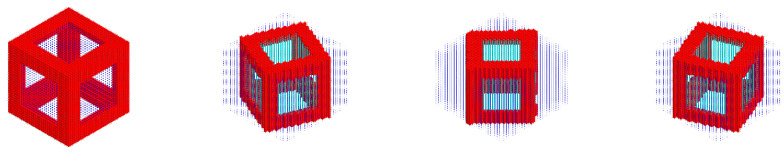
A grid lattice cell discretized by 30 voxels along the x, y, and z axis, where the unit cell rotated at 0°, 22°, 45°, and 68° along the z-axis.

### 3.4. Numerical Errors Due to Approximating Periodicity

**Figure 14:**
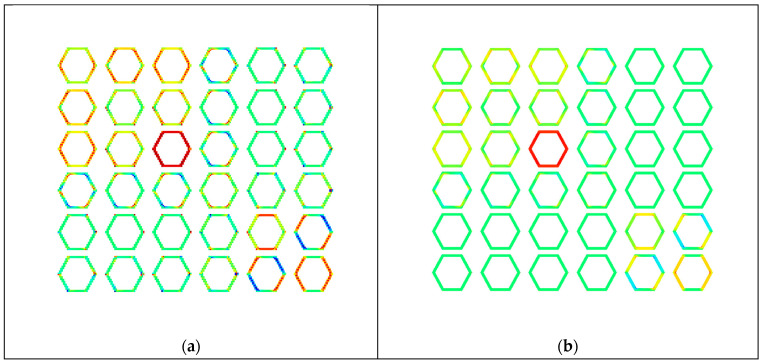
Visualization of the integral in Equation (18) for individual voxel elements, a hexagon with $l_{x}/l_{y}=1.155$ and a voxel shape of (**a**) 30×30×1 and (**b**) 150×150×1. Each of the individual hexagon visualizations were normalized by the value of the $C_{ij}^{H}$ term.

In this section, a hexagon was meshed with an equal number of divisions along the global x and y axes and one element along the z-axis. The following term ($\tau_{e}$) has been visualized in Figure 14:

(18) $$\tau_{e}^{\left(i\right)\left(j\right)}=\frac{1}{C_{ij}^{H}}\int_{V_{e}}{\left(\chi_{e}^{0\left(i\right)}-\chi_{e}^{\left(i\right)}\right)}^{T}k_{e}\left(\chi_{e}^{0\left(j\right)}-\chi_{e}^{\left(j\right)}\right)dV_{e}$$

where $\chi_{e}^{0\left(i\right)}$ and $\chi_{e}^{\left(i\right)}$ are the element’s macroscopic and microscopic displacement due to unit strain i, and $\tau_{e}$ represents the contribution of element e to the homogenized elastic tensor $C_{ij}^{H}$ normalized by the total $C_{ij}^{H}$. By visualizing $\tau_{e}$ for each element, it is possible to observe the elements that could cause the numerical errors shown in Figure 11. The colour scheme used in Figure 11 is such that the maximum absolute value dictates the positive (red) and negative (blue) limits of the plot. Values close to zero are given a green colour.

For the hexagon closed topology with a volume fraction of 0.29 using isotropic steel with $E_{iso}={2.0\times 10}^{11}$ and $\nu_{iso}=0.3$, which has been discretized as 30×30×1 voxels ($l_{x}/l_{y}=1.155$), the homogenized elastic tensor was computed to be:

(19) $$\left[C_{ij}^{H}\right]=\left[\begin{array}{cccccc} 23.8422 & 18.3845 & 12.5175 & -0.0127 & -0.0589 & -0.1269\\ 18.3845 & 23.0329 & 12.3423 & -0.0116 & -0.0204 & 0.0223\\ 12.5175 & 12.3423 & 72.047 & -0.0278 & -0.0421 & -0.0378\\ -0.0127 & -0.0116 & -0.0278 & 3.3903 & -0.0154 & 0.0016\\ -0.0589 & -0.0204 & -0.0421 & -0.0154 & 13.0261 & -0.0147\\ -0.1269 & 0.0223 & -0.0378 & 0.0016 & -0.0147 & 14.7842 \end{array}\right]\times 10^{10}$$

After the mesh resolution was increased to 150×150×1 voxels ($l_{x}/l_{y}=1.155$), with a volume fraction of 0.30, the refined, homogenized elastic tensor was computed to be:

(20) $$\left[C_{ij}^{H}\right]=\left[\begin{array}{cccccc} 24.6521 & 18.4319 & 12.9212 & 0.0001 & 0.0001 & -0.0009\\ 18.4319 & 24.5085 & 12.8797 & -0.0001 & 0.0002 & 0.0014\\ 12.9212 & 12.8797 & 71.0627 & -0.0001 & 0.0004 & 0.0006\\ 0.0001 & -0.0001 & -0.0001 & 3.2672 & -0.0004 & 0.0001\\ 0.0001 & 0.0002 & 0.0004 & -0.0004 & 14.020 & -0.0002\\ -0.0009 & 0.0014 & 0.0006 & 0.0001 & -0.0002 & 14.1044 \end{array}\right]\times 10^{10}$$

**Figure 15:**
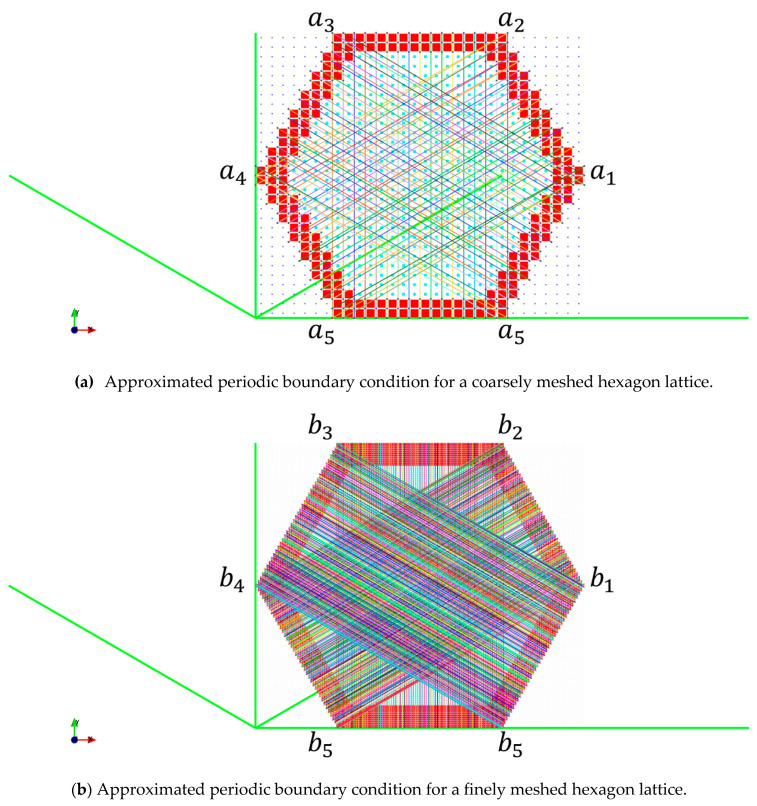
Visualization of the periodicity node pairs. Each line of a random colour represents a coupling of the degrees of freedom for that node pair. The periodic basis has been highlighted as thick green lines. The approximated periodic boundary condition can be observed while comparing how the periodic boundary condition is applied at (**a**) between regions a_1_ to a_5_ and a_2_ to a_4_. Due to the finer mesh size, the approximation of the periodic boundary condition at (**b**) is not affected.

The contributions of the voxels that contribute the small non-zero terms for the 30×30×1 and the 150×150×1 are shown in Figure 14a b, respectively. The comparison of the eigen values with and without the zero terms is tabulated in Table 1. Comparing the hexagon corresponding to $C_{51}^{H}$ in Figure 14a with Figure 15a, it can be seen that the voxels that have the highest contributions belong to the group whose periodicity has not been matched properly due to discretizing the periodicity basis vector; this can be seen in Figure 15a—the line that marks the periodic node pair for the nodes near the marked area of $a_{1}$ to $a_{5}$, is not parallel with the periodic basis (bold green line), and a single node from $a_{1}$ is periodic, with two nodes from $a_{5}$ zone. This behaviour causes over-stiffening of the nodal degree of freedom because the stiffness corresponding to another node would be accumulated into one node during the global stiffness formulation. Similarly, if the nodes on the cell envelope are not periodically matched with another node on the envelope, it would cause the stiffness of the nodal degree of freedom to be smaller than the ones that have been matched. The over-stiffening and the under-stiffening impact the microscopic displacements, which contribute to values that are above or below the actual threshold. The mismatch in the periodic degrees of freedom would remove planes of symmetry and would cause the RVE with hexagonal symmetry to be represented as a monoclinic or a triclinic material, as shown in Figure 9.

**Figure 16:**
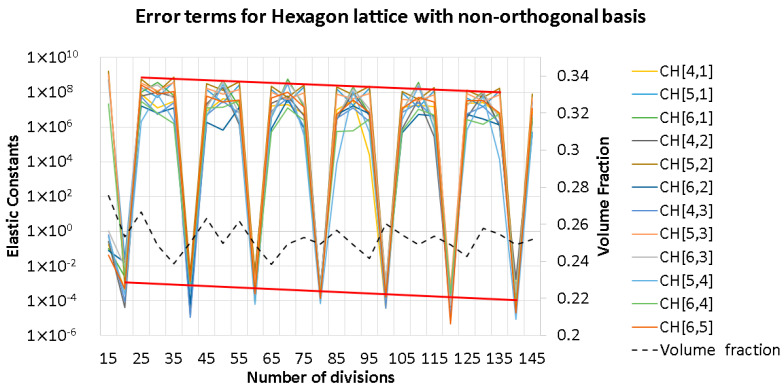
Convergence of error terms for the hexagon lattice with non-unity aspect ratio. The datum shown above is a reduced subset of Figure 7c to emphasize the decreasing residual errors (thick red line).

Furthermore, it can be observed from Figure 14 and Figure 16 that increasing the voxel discretization reduces the small non-zero components in the $\left[C_{ij}^{H}\right]$ tensor because each voxel contributes a smaller value due to the smaller voxel volume. In Figure 14, for the hexagon with a larger voxel size, the residual terms are contributed by the voxels along the edges caused by imperfect periodic boundary conditions; this is also the case for the refined hexagon lattice, but the ratio of voxels in the edge is smaller than the hexagon with larger voxel size, so the contribution of the residual error is decreased.

### 3.5. Comparison of Young’s Modulus with Volume Fraction

**Figure 17:**
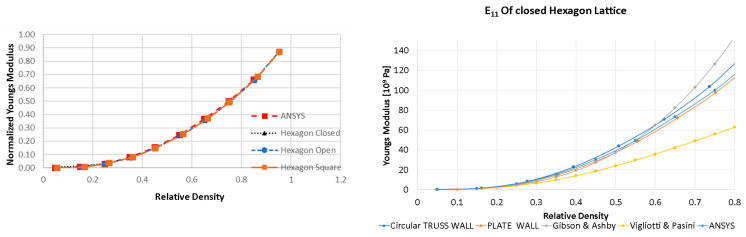
Comparison of normalized $E_{11}$ for the hexagon lattice at multiple volume fractions across multiple homogenization schemes present in the literature [26,36].

**Figure 18:**
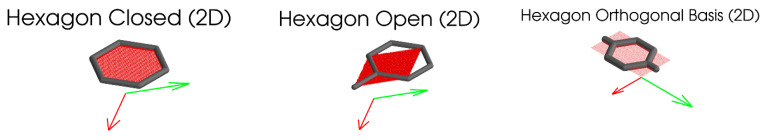
Multiple representations of the Honeycomb hexagon lattice with different representative volume elements with their corresponding periodic basis. The representative volume element of the unit cell is shown as red voxels.

The $E_{11}$ elastic property for the hexagon lattice that is obtained from the voxelization code and ANSYS are compared with different homogenization procedures obtained from Gibson and Ashby [36] and Vigliotti and Pasini [26]. In Figure 17, the $E_{11}$, properties calculated from the voxelized closed cell hexagon, open cell hexagon, and hexagon cell envelope (all shown in Figure 18) with orthogonal basis are compared. No significant deviations were observed when comparing it to the results from ANSYS, which were obtained from a hexagon cell envelope with an orthogonal basis.

A hexagon lattice made from a circular truss (i.e., without considering periodicity in the out-of-plane direction) and a hexagon with a plate wall (3D voxel with a single layer and periodicity in the z-direction) are considered below. Gibson and Ashby’s model [36] starts to overpredict the elasticity, whereas Vigliotti and Pasini’s [26] model, which uses a beam model, underpredicts the elasticity at higher relative density.

### 3.6. Comparison of Elastic Properties for Hexagon Lattice with Varying Cell Angle

**Figure 19:**
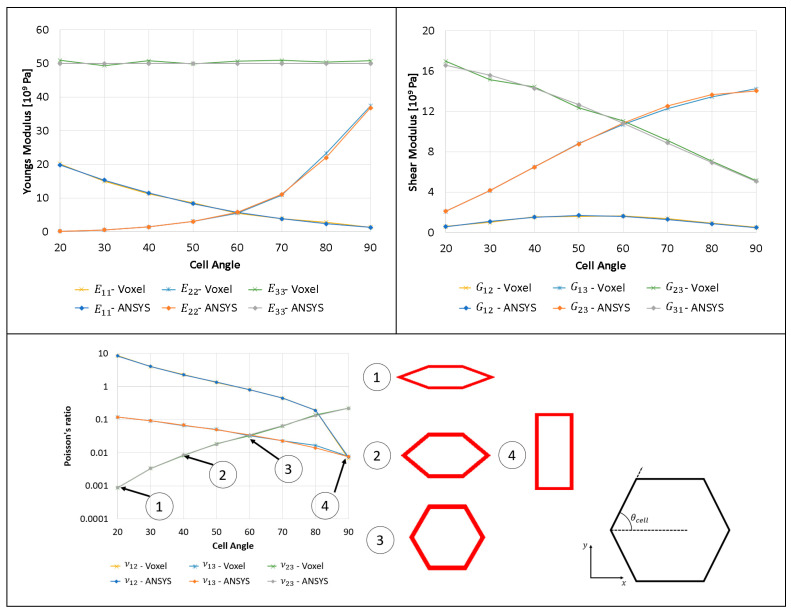

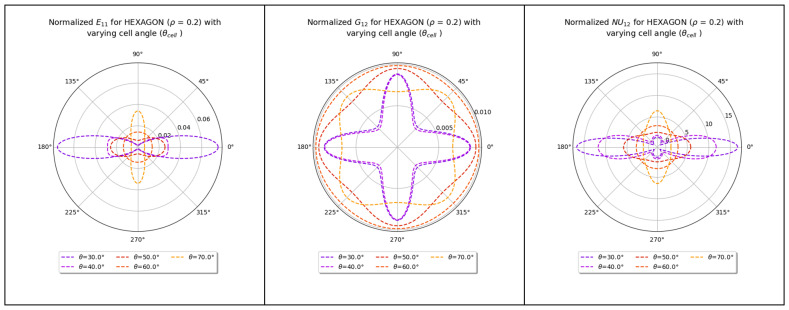
Elastic properties of the 2D hexagon lattice with a relative density of 0.25 made from isotropic material with Young’s modulus of 2 × 10^11^ Pa and Poisson’s ratio of 0.3. Two-dimensional anisotropic diagram of elastic properties for the hexagon with a volume fraction of 0.2 due to the variation of the hexagon cell angle.

In this section, the cell angle is changed, and Young’s modulus and shear modulus are plotted for a hexagon with a relative density of 0.2. The results obtained from the voxel homogenization process match very well, with some slight discrepancies; this is caused by the geometry discretization process, where a volume fraction of 0.2 was not achieved due to the linear interpolation used with the optimizer to define the relative density as a function of the radius of the truss elements. The variation of the homogenized elastic properties for the hexagon lattice w.r.t cell angle is presented in Figure 19.

### 3.7. Comparison of Elastic Constants for a 2D Monoclinic RVE Lattice with Varying Cell Angle

**Figure 20:**
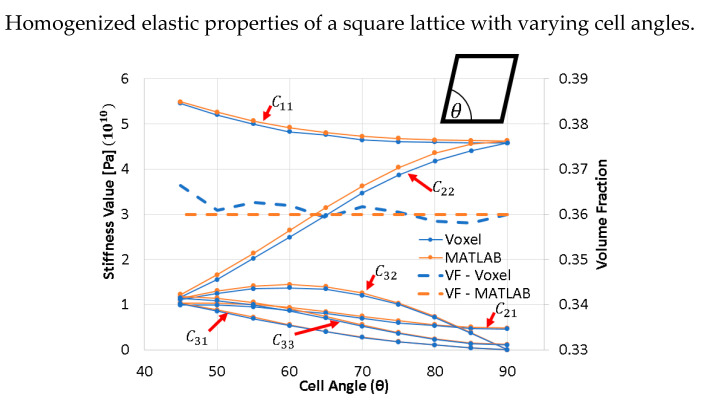
Comparison of homogenized elastic properties of a square lattice with varying cell angles between 2D MATLAB voxel and 3D voxel code.

In this section, a square-like 2D lattice is considered; the angle shown varies from 45° to 90°. The results are compared with code published by Andreassen and Andreasen [33] as shown in Figure 20. Andreassen’s work is considered more accurate because the 2D voxel elements change shape as the cell angle changes, and there are no approximations performed when applying periodicity boundary conditions to the lattice. The voxel code underestimates the $C_{22}$ component of the stiffness matrix because of the periodicity approximations and the voxel meshing algorithm. The main cause is the voxel meshing, where the truss elements are jagged, as shown in Figure 8.

## 4. Application

In this section, application of the developed voxel code is applied to different lattice topologies with non-orthogonal bases. The material used in this study is isotropic steel with $E_{iso}={2.0\times 10}^{11}$ Pa and $\nu_{iso}=0.3$.

### 4.1. Three-Dimensional Triclinic and a Monoclinic Bravais Grid Lattice

**Figure 21:**
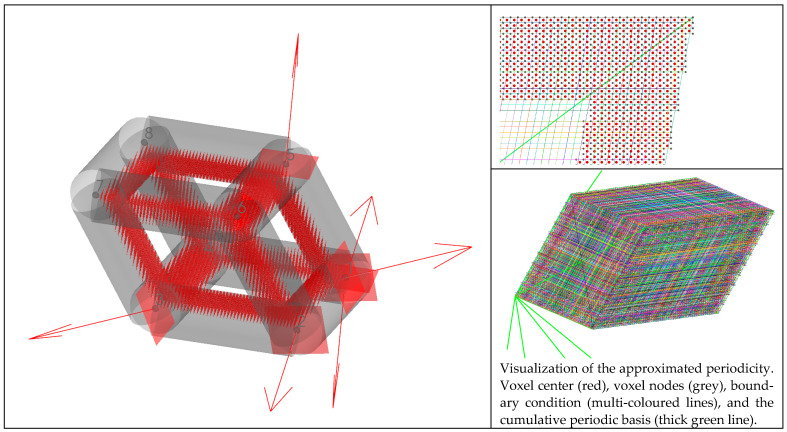
Discretized triclinic Bravais grid lattice. The Voxel center is represented as a small sphere for a volume fraction of 0.3. The planes that represent the cell envelope definition and its normal have also been plotted.

In this section, a triclinic and a monoclinic Bravais grid lattice are analyzed by applying the approximated periodicity boundary condition. The geometry was discretized using voxels, as shown in Figure 21.

**Figure 22:**
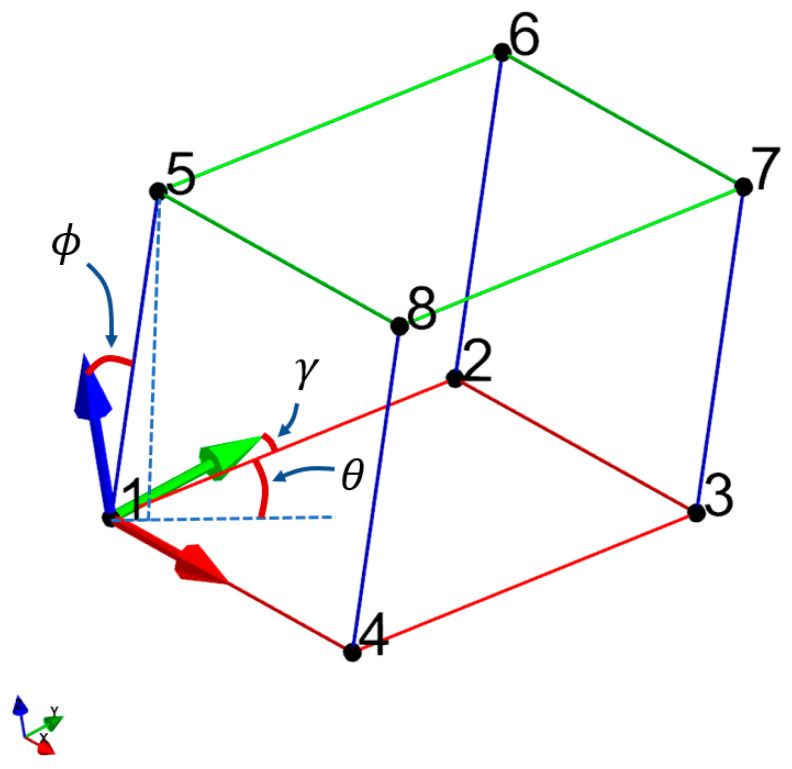
Base lattice definition for monoclinic and triclinic Bravais lattice.

The lattice definition used for the current analysis to create the triclinic and monoclinic Bravais grid lattice is shown in Figure 22. The lattice is defined such that the red trusses are in the global x–y plane. The trusses $T_{14}$ and $T_{23}$ are aligned parallel to the global x-axis. The other red trusses $T_{12}$ and $T_{43}$, are rotated from the global y-axis using angle gamma (γ) in the x–y plane. The green trusses are created by offsetting the red trusses using the blue trusses. The blue trusses are defined using a spherical coordinate system with ϕ and θ rotation angles, as shown in Figure 22. The periodic basis for the lattice was defined using the trusses $T_{12}$, $T_{14}$, and $T_{15}$. The angular restrictions for ϕ, θ, and γ for the Bravais lattice system are tabulated in Table 2.

The key difference between the triclinic and the monoclinic Bravais grid lattice used for the current analysis is the slight change in the truss angles and their corresponding periodicity bases. It can be noted that for the monoclinic lattice, the trusses that make up the red and the green frames are orthogonal to each other (γ=0°). Furthermore, the point set {1,4,5,8} and {2,3,6,7} are co-planar with the z–x plane.

**Figure 23:**
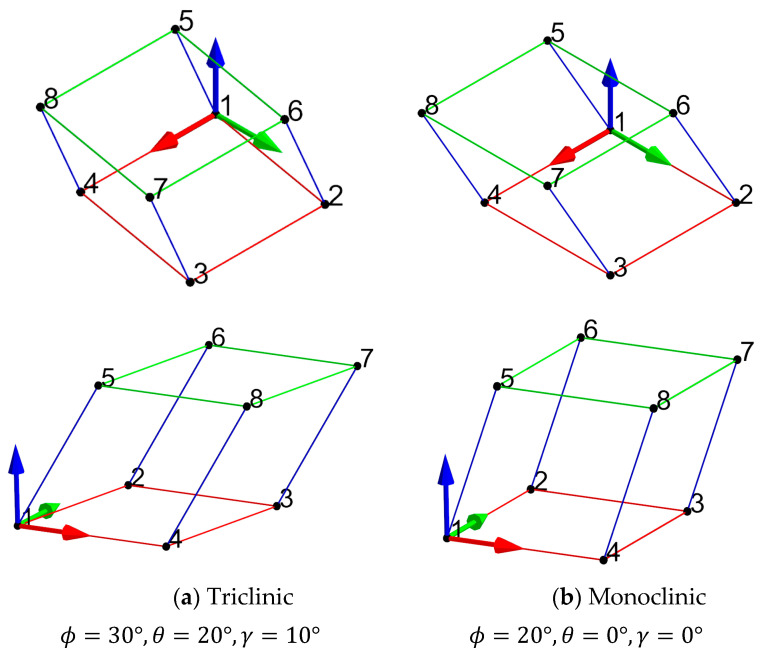
Geometric definitions for the monoclinic and triclinic grid lattice are used in this paper.

For the monoclinic Bravais grid lattice shown in Figure 23b, which had a volume fraction of 0.29 and was discretized as 30 × 30 × 1 voxels, the homogenized elastic tensor was computed to be:

(21) $$\left[C_{ij}^{H}\right]=\left[\begin{array}{cccccc} 31.0421 & 3.8754 & 5.0174 & 0.0000 & 0.0000 & 1.9074\\ & 30.0581 & 2.9965 & 0.0000 & 0.0000 & 1.2017\\ & & 24.1917 & 0.0000 & 0.0000 & 6.9772\\ & & & 2.7767 & 0.5141 & 0.0000\\ & & & & 2.4535 & 0.0000\\ & & & & & 5.2315 \end{array}\right]\times 10^{9}$$

Similarly, for the triclinic Bravais grid lattice shown in Figure 23a and Figure 23, which had a volume fraction of 0.29 and was discretized as 30 × 30 × 1 voxels, the homogenized elastic tensor was computed to be

(22) $$\left[C_{ij}^{H}\right]=\left[\begin{array}{cccccc} 31.8431 & 4.3748 & 5.2469 & 1.3106 & 1.1570 & 2.8994\\ & 27.8371 & 2.3521 & 4.1020 & 0.3740 & 1.3446\\ & & 18.1782 & 1.6143 & 2.6998 & 7.1696\\ & & & 3.5984 & 0.8139 & 1.2457\\ & & & & 2.5514 & 1.3011\\ & & & & & 6.3248 \end{array}\right]\times 10^{9}$$

**Figure 24:**
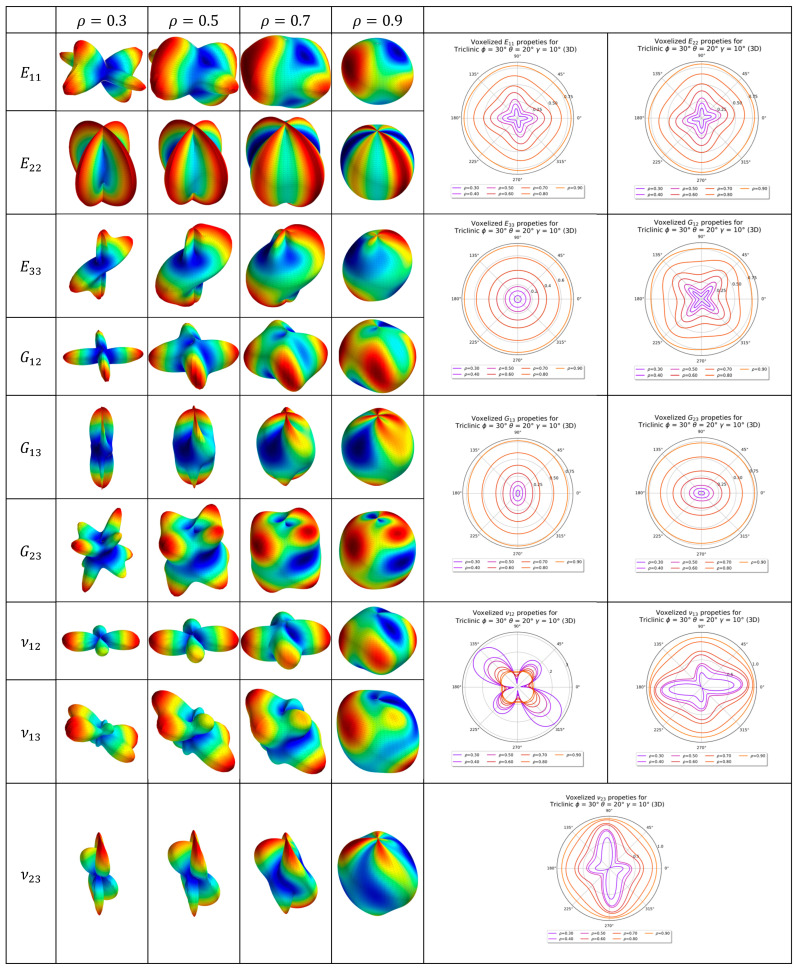
Two-dimensional and three-dimensional anisotropy plots for triclinic Bravais grid lattice (3D). Three-dimensional anisotropy plots are not to scale.

**Figure 25:**
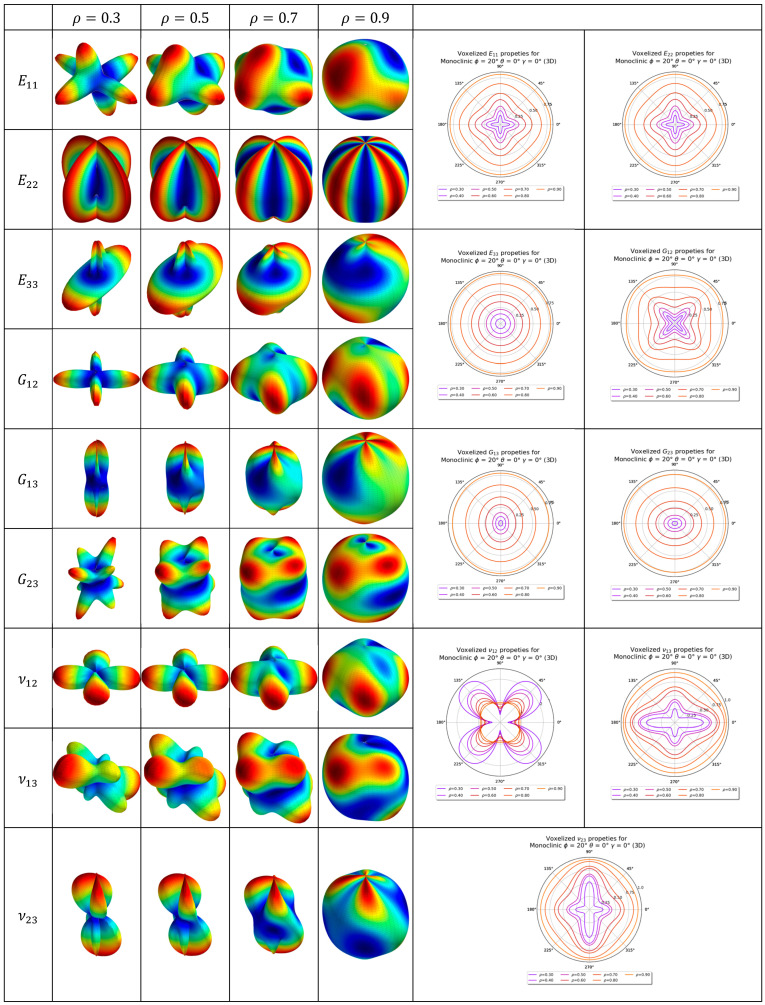
Two-dimensional and three-dimensional anisotropy plots for monoclinic Bravais grid lattice (3D). Three-dimensional anisotropy plots are not to scale.

The elastic properties of the monoclinic and the triclinic lattice are tabulated in Table 3. The evolution of the elastic properties visualized as 2D and 3D anisotropic plots is shown in Figure 24 and Figure 25 for the triclinic and monoclinic Bravais grid lattice, respectively. The key difference between the 3D anisotropy plot between the triclinic and monoclinic Bravais grid lattice is how the peaks of the anisotropy plots are warped.

### 4.2. Two-Dimensional Non-Orthogonal Lattice

**Figure 26:**
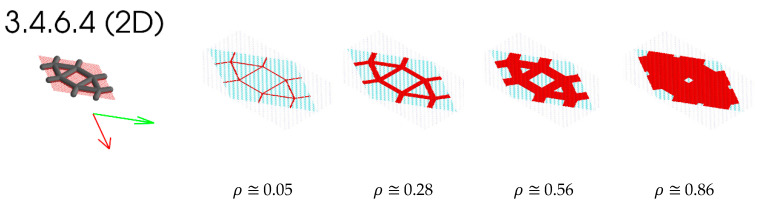
3.4.6.4 lattice cells with a non-orthogonal periodic basis. The representative volume element of the unit cell is shown as red voxels. Evolution of selected 2D latices as the volume fraction increases.

In this section, the elastic properties of the 3.4.6.4 2D lattice are plotted. The homogenized elastic tensor of the 3.4.6.4 lattices shown in Figure 26 is tabulated in Table 3. The tabulated tensor is based on voxels that are generated as a single layer with an additional periodicity added out of a plane (z-axis). Considering this periodicity means that the single layer is equivalent to a fully tessellated geometry that has infinite depth in the out-of-plane direction. The tabulated tensors are obtained by fitting a third-order polynomial for each of the tensor entries, where the volume fraction varies from 0.1 to 0.9.

**Figure 27:**
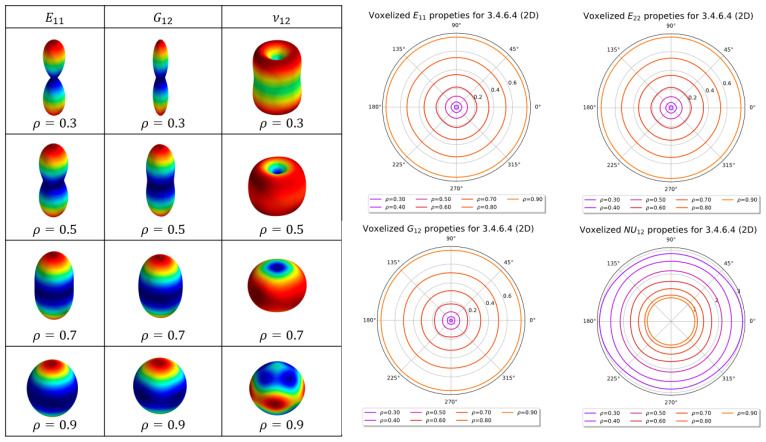
Two-dimensional and three-dimensional anisotropy plots for 3.4.6.4 lattice.

Figure 26 depicts the 3.4.6.4 lattice for multiple volume fractions. This figure shows how the geometry of the lattice changes as the volume fraction increases. This sort of filling behaviour is one of the reasons for the Euler–Bernoulli beam formulation being invalid for larger relative density, as it cannot predict the interactions of the geometry within the lattice. The 2D and 3D anisotropy plots for the 3.4.6.4 lattice are shown in Figure 27. The coefficients for the homogenized elastic tensor as a function of relative density is tabulated in Table 4.

### 4.3. Three-Dimensional Sandwich Panel Lattice

**Figure 28:**
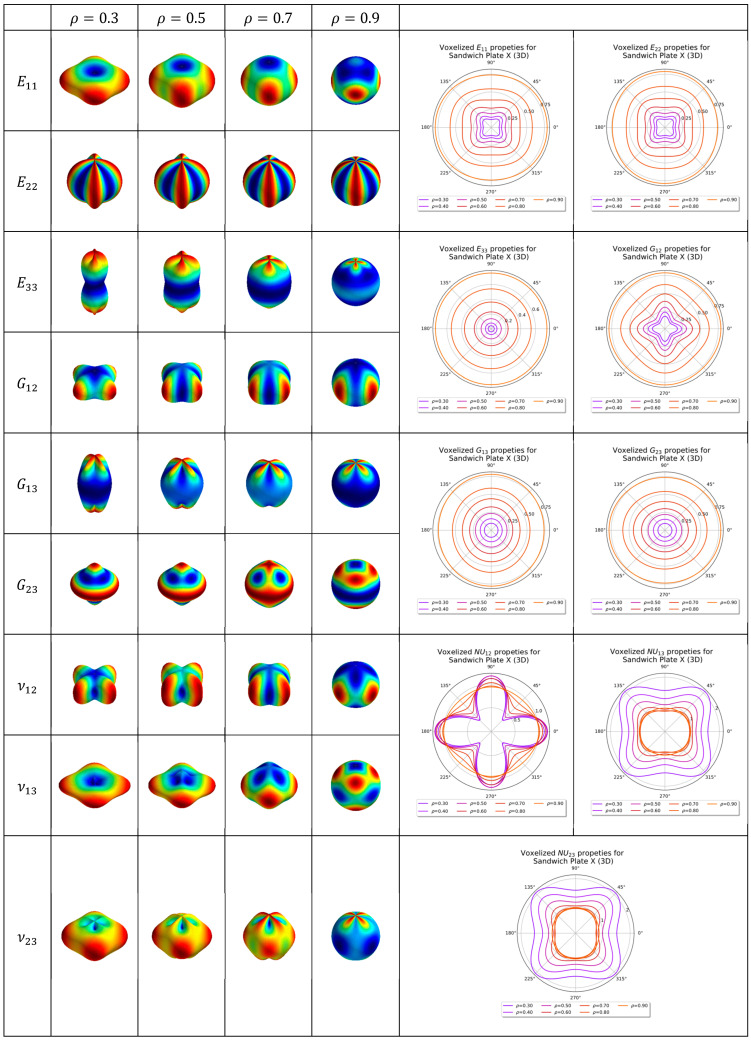
Two-dimensional and three-dimensional anisotropy plots for X lattice in a sandwich panel (3D).

In this section, the elastic properties of the sandwiched X (body-centred cubic) lattice are plotted. For the sandwich panel analysis, periodicity in the z-direction (sandwich plate normal) is assumed; thus, the elastic properties that are plotted in this section are for sandwich panels that are assumed to be stacked. For all the sandwich panel geometries analyzed in this paper, the thickness of the sandwich panel is held constant at a unit of 0.05 of the cell length. The homogenized elastic tensor of the Sandwich X lattice is shown and tabulated in Table 3. The 2D and 3D anisotropy plots for the Sandwich X lattice are shown in Figure 28. The coefficients for the homogenized elastic tensor as a function of relative density is tabulated in Table 5.

## 5. Conclusions

The homogenized elastic properties of several 2D and 3D lattices can be performed using the voxel mesh approach regardless of whether the periodicity is orthogonal or non-orthogonal. Approximating the periodicity on an imperfect voxel mesh misaligns the periodic node pairs; this alters or removes the planes of symmetry within the RVE and introduces errors in the stiffness matrix. These errors are at least two orders of magnitude smaller than the main diagonals for the imperfect voxel mesh. The mesh is considered perfectly voxelized if the translated nodes of the cell envelope, using the periodicity basis, align themselves with another node. For this perfectly voxelized mesh, the numerical errors are at least eight orders of magnitude smaller than the main diagonals; this can be achieved by altering the number of discretizations performed along the global coordinate system. Furthermore, the numerical error caused by approximating the non-orthogonal periodicity decreases as the voxel size is reduced. We have shown that it is possible to evaluate the elastic properties of periodic cellular materials whose periodic basis is of any Bravais lattice system.

## Figures and Tables

**Table 1:** Comparison of eigenvalues of lower resolution (19) and higher resolution (20) hexagon lattice for an imperfectly voxelized hexagon with a voxel aspect ratio of 1.155.

Eigenvalues of (19) (10^11^)	Eigenvalues of (19) with Zeros (10^11^)	% Diff.	Eigenvalues of (20) (10^11^)	Eigenvalues of (20) with Zeros (10^11^)	% Diff.	% Diff. Between (19) and (20)
8.0118	8.0117	0.0002	8.0050	8.0050	0	0.08
3.3757	3.3757	0.0006	3.4025	3.4025	0	−0.79
1.4785	1.4784	0.0070	1.4104	1.4104	0	4.8
1.3026	1.3026	−0.0009	1.4020	1.4020	0	−7.1
0.5047	0.5048	−0.0241	0.6148	0.6148	0	−17.94
0.3390	0.3390	−0.0012	0.3267	0.3267	0	3.77

**Table 2:** Angular constraints for the Bravais lattice are based on the angle definitions used in this paper.

	Phi (ϕ)	Theta (θ)	Gamma (γ)
Triclinic (ϕ≠θ≠γ)	≠0°	≠0°	≠0°
Monoclinic	≠0°	=0°	=0°
Cubic, Orthorhombic, and Tetragonal	=0°	=0°	=0°

**Table 3:** Elastic properties of the sample monoclinic and triclinic Bravais lattice.

Elastic Properties	Monoclinic	Triclinic
$E_{11}$ [Pa]	2.96 × 10^10^	2.96 × 10^10^
$E_{22}$ [Pa]	2.93 × 10^10^	2.27 × 10^10^
$E_{33}$ [Pa]	1.46 × 10^10^	9.28 × 10^9^
$G_{12}$ [Pa]	2.67 × 10^9^	2.68 × 10^9^
$G_{13}$ [Pa]	2.36 × 10^9^	2.03 × 10^9^
$G_{23}$ [Pa]	3.21 × 10^9^	3.36 × 10^9^
$\nu_{12}$	0.108	0.1247
$\nu_{13}$	0.156	0.1668
$\nu_{23}$	0.076	0.0778

**Table 4:** Homogenized elastic tensor coefficients (based on Equation (13)) and correlation coefficient (R) for the polynomial fit for the 3.4.6.4 lattice.

		$C_{11}$	$C_{22}$	$C_{33}$	$C_{44}$	$C_{55}$	$C_{66}$	$C_{12}$	$C_{13}$	$C_{23}$
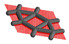 3.4.6.4 (2D)	$\rho^{3}$	1.591	1.644	0.377	0.538	0.210	0.178	0.476	0.620	0.636
	$\rho^{2}$	−0.662	−0.731	−0.189	−0.136	−0.014	0.026	−0.351	−0.304	−0.325
	ρ	0.380	0.390	1.132	0.011	0.190	0.180	0.347	0.218	0.221
	R	0.999	0.999	1.000	1.000	1.000	1.000	0.995	0.999	0.999

**Table 5:** Homogenized elastic tensor coefficients (based on Equation (13)) and correlation coefficient ($R^{2}$) for the polynomial fit for the Sandwich X lattice.

		$C_{11}$	$C_{22}$	$C_{33}$	$C_{44}$	$C_{55}$	$C_{66}$	$C_{12}$	$C_{13}$	$C_{23}$
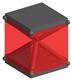 Sandwich X Lattice (3D)	$\rho^{3}$	2.318	2.318	1.499	0.456	0.127	0.127	0.78	0.607	0.607
	$\rho^{2}$	−1.915	−1.915	−0.296	−0.387	0.193	0.193	−0.646	−0.265	−0.265
	ρ	0.941	0.941	0.118	0.32	0.045	0.045	0.365	0.14	0.14
	$R^{2}$	0.999	0.999	1.0	0.999	0.999	0.999	0.998	0.995	0.995

## Data Availability

The data that support the findings of this study are available on request from the corresponding author.

## Supplementary Materials

The following supporting information can be downloaded at: https://www.mdpi.com/article/10.3390/ma16247562/s1.

## References

[1] Tancogne-Dejean T., Spierings A.B., Mohr D. (2016). Additively-Manufactured Metallic Micro-Lattice Materials for High Specific Energy Absorption under Static and Dynamic Loading. Acta Mater..

[2] Ling B., Wei K., Wang Z., Yang X., Qu Z., Fang D. (2020). Experimentally Program Large Magnitude of Poisson’s Ratio in Additively Manufactured Mechanical Metamaterials. Int. J. Mech. Sci..

[3] Wang C., Gu X., Zhu J., Zhou H., Li S., Zhang W. (2020). Concurrent Design of Hierarchical Structures with Three-Dimensional Parameterized Lattice Microstructures for Additive Manufacturing. Struct. Multidiscip. Optim..

[4] Chen L.-Y., Liang S.-X., Liu Y., Zhang L.-C. (2021). Additive Manufacturing of Metallic Lattice Structures: Unconstrained Design, Accurate Fabrication, Fascinated Performances, and Challenges. Mater. Sci. Eng. R Rep..

[5] Chen Y., Zhao B., Liu X., Hu G. (2020). Highly Anisotropic Hexagonal Lattice Material for Low Frequency Water Sound Insulation. Extrem. Mech. Lett..

[6] Yin S., Wu L., Yang J., Ma L., Nutt S. (2014). Damping and Low-Velocity Impact Behavior of Filled Composite Pyramidal Lattice Structures. J. Compos. Mater..

[7] Murray G., Gandhi F., Hayden E. (2012). Polymer-Filled Honeycombs to Achieve a Structural Material with Appreciable Damping. J. Intell. Mater. Syst. Struct..

[8] Yang R., Yang Q., Niu B. (2020). Design and Study on the Tailorable Directional Thermal Expansion of Dual-Material Planar Metamaterial. Proc. Inst. Mech. Eng. Part C J. Mech. Eng. Sci..

[9] Xu H., Farag A., Pasini D. (2018). Routes to Program Thermal Expansion in Three-Dimensional Lattice Metamaterials Built from Tetrahedral Building Blocks. J. Mech. Phys. Solids J. Homepage.

[10] Wei K., Chen H., Pei Y., Fang D. (2016). Planar Lattices with Tailorable Coefficient of Thermal Expansion and High Stiffness Based on Dual-Material Triangle Unit. J. Mech. Phys. Solids.

[11] Nelissen W.E.D., Ayas C., Tekõ Glu C. (2019). 2D Lattice Material Architectures for Actuation. J. Mech. Phys. Solids J. Homepage.

[12] McHale C., Telford R., Weaver P.M. (2020). Morphing Lattice Boom for Space Applications. Compos. Part B Eng..

[13] Li M.Z., Stephani G., Kang K.J. (2011). New Cellular Metals with Enhanced Energy Absorption: Wire-Woven Bulk Kagome (WBK)-Metal Hollow Sphere (MHS) Hybrids. Adv. Eng. Mater..

[14] Murray G.J., Gandhi F. (2013). Auxetic Honeycombs with Lossy Polymeric Infills for High Damping Structural Materials. J. Intell. Mater. Syst. Struct..

[15] Tao Y., Duan S., Wen W., Pei Y., Fang D. (2017). Enhanced Out-of-Plane Crushing Strength and Energy Absorption of in-Plane Graded Honeycombs. Compos. Part B Eng..

[16] Dinovitzer M., Miller C., Hacker A., Wong G., Annen Z., Rajakareyar P., Mulvihill J., El Sayed M. (2019). Structural Development and Multiscale Design Optimization of Additively Manufactured UAV with Blended Wing Body Configuration Employing Lattice Materials. Proceedings of the AIAA Scitech 2019 Forum.

[17] Zhu J.H., Zhang W.H., Xia L. (2016). Topology Optimization in Aircraft and Aerospace Structures Design. Arch. Comput. Methods Eng..

[18] Askar A., Cakmak A.S.S. (1968). A Structural Model of a Micropolar Continuum. Int. J. Eng. Sci..

[19] Chen J.Y., Huang Y., Ortiz M. (1998). Fracture Analysis of Cellular Materials: A Strain Gradient Model. J. Mech. Phys. Solids.

[20] Bažant Z.P., Christensen M. (1972). Analogy between Micropolar Continuum and Grid Frameworks under Initial Stress. Int. J. Solids Struct..

[21] Kumar R.S., McDowell D.L. (2004). Generalized Continuum Modeling of 2-D Periodic Cellular Solids. Int. J. Solids Struct..

[22] van Tuijl R.A., Remmers J.J.C., Geers M.G.D. (2019). Multi-Dimensional Wavelet Reduction for the Homogenisation of Microstructures. Comput. Methods Appl. Mech. Eng..

[23] Vigliotti A., Pasini D. (2013). Mechanical Properties of Hierarchical Lattices. Mech. Mater..

[24] Hutchinson R.G., Fleck N.A. (2006). The Structural Performance of the Periodic Truss. J. Mech. Phys. Solids.

[25] Elsayed M.S.A., Pasini D. (2010). Analysis of the Elastostatic Specific Stiffness of 2D Stretching-Dominated Lattice Materials. Mech. Mater..

[26] Vigliotti A., Pasini D. (2012). Linear Multiscale Analysis and Finite Element Validation of Stretching and Bending Dominated Lattice Materials. Mech. Mater..

[27] Florence C., Sab K. (2006). A Rigorous Homogenization Method for the Determination of the Overall Ultimate Strength of Periodic Discrete Media and an Application to General Hexagonal Lattices of Beams. Eur. J. Mech. A/Solids.

[28] Assidi M., Dos Reis F., Ganghoffer J.-F. (2011). Equivalent Mechanical Properties of Biological Membranes from Lattice Homogenization. J. Mech. Behav. Biomed. Mater..

[29] Dos Reis F., Ganghoffer J.F. (2012). Equivalent Mechanical Properties of Auxetic Lattices from Discrete Homogenization. Comput. Mater. Sci..

[30] Hassani B., Hinton E. (1998). A Review of Homogenization and Topology Optimization I—Homogenization Theory for Media with Periodic Structure. Comput. Struct..

[31] Hollister S.J., Kikuehi N. (1992). A Comparison of Homogenization and Standard Mechanics Analyses for Periodic Porous Composites. Comput. Mech..

[32] Guedes J.J.M.J., Kikuchi N. (1990). Preprocessing and Postprocessing for Materials Based on the Homogenization Method with Adaptive Finite Element Methods. Comput. Methods Appl. Mech. Eng..

[33] Andreassen E., Andreasen C.S. (2014). How to Determine Composite Material Properties Using Numerical Homogenization. Comput. Mater. Sci..

[34] Dong G., Tang Y., Zhao Y.F. (2019). A 149 Line Homogenization Code for Three-Dimensional Cellular Materials Written in MATLAB. J. Eng. Mater. Technol. Trans. ASME.

[35] Masters I.G., Evans K.E. (1996). Models for the Elastic Deformation of Honeycombs. Compos. Struct..

[36] Gibson L.J., Ashby M.F., Gibson L.J., Ashby M.F. (1997). Cellular Solids: Structure and Properties.

[37] Christensen R.M. (2000). Mechanics of Cellular and Other Low-Density Materials. Int. J. Solids Struct..

[38] Elsayed M.S.A. (2010). Multiscale Mechanics and Structural Design of Periodic Cellular Materials.

[39] Vigliotti A., Pasini D. (2012). Stiffness and Strength of Tridimensional Periodic Lattices. Comput. Methods Appl. Mech. Eng..

[40] Kalamkarov A.L., Andrianov I.V., Danishevs’kyy V.V. (2009). Asymptotic Homogenization of Composite Materials and Structures. Appl. Mech. Rev..

[41] Masoumi E., Abad K., Khanoki S.A., Pasini D. (2012). Fatigue Design of Lattice Materials via Computational Mechanics: Application to Lattices with Smooth Transitions in Cell Geometry. Int. J. Fatigue.

[42] Cheng G.D., Cai Y.W., Xu L. (2013). Novel Implementation of Homogenization Method to Predict Effective Properties of Periodic Materials. Acta Mech. Sin. Xuebao.

[43] Liu P., Liu A., Peng H., Tian L., Liu J., Lu L. (2021). Mechanical Property Profiles of Microstructures via Asymptotic Homogenization. Comput. Graph..

[44] Arabnejad S., Pasini D. (2013). Mechanical Properties of Lattice Materials via Asymptotic Homogenization and Comparison with Alternative Homogenization Methods. Int. J. Mech. Sci..

[45] Peng X., Cao J. (2002). A Dual Homogenization and Finite Element Approach for Material Characterization of Textile Composites. Compos. Part B Eng..

[46] Guinovart-Daz R., Rodrguez-Ramos R., Bravo-Castillero J., Sabina F.J., Dario Santiago R., Martinez Rosado R. (2007). Asymptotic Analysis of Linear Thermoelastic Properties of Fiber Composites. J. Thermoplast. Compos. Mater..

[47] Visrolia A., Meo M. (2013). Multiscale Damage Modelling of 3D Weave Composite by Asymptotic Homogenisation. Compos. Struct..

[48] Takano N., Zako M., Kubo F., Kimura K. (2003). Microstructure-Based Stress Analysis and Evaluation for Porous Ceramics by Homogenization Method with Digital Image-Based Modeling. Int. J. Solids Struct..

[49] Matsui K., Terada K., Yuge K. (2004). Two-Scale Finite Element Analysis of Heterogeneous Solids with Periodic Microstructures. Comput. Struct..

[50] Omairey S.L., Dunning P.D., Sriramula S. (2019). Development of an ABAQUS Plugin Tool for Periodic RVE Homogenisation. Eng. Comput..

[51] Material Designer User’s Guide. https://ansyshelp.ansys.com/account/secured?returnurl=/Views/Secured/corp/v194/acp_md/acp_md.html.

[52] Barbero E.J. (2013). Finite Element Analysis of Composite Materials Using AbaqusTM..

[53] Hassani B., Hinton E. (1998). A Review of Homogenization and Topology Opimization II—Analytical and Numerical Solution of Homogenization Equations. Comput. Struct..

[54] Hassani B., Hinton E. (1998). A Review of Homogenization and Topology Optimization III—Topology Optimization Using Optimality Criteria. Comput. Struct..

[55] Rajakareyar P. (2023). Thermo-Elastic Mechanics of Morphing Lattice Structures with Applications in Shape Optimization of BLI Engine Intakes.

[56] Virtanen P., Gommers R., Oliphant T.E., Haberland M., Reddy T., Cournapeau D., Burovski E., Peterson P., Weckesser W., Bright J. (2020). SciPy 1.0: Fundamental Algorithms for Scientific Computing in Python. Nat. Methods.

[57] Kermode J. Lars Pastewka Matscipy/Elasticity.Py at Master LibAtoms/Matscipy. https://github.com/libAtoms/matscipy/blob/master/matscipy/elasticity.py.

[58] Van Der Walt S., Colbert S.C., Varoquaux G. (2011). The NumPy Array: A Structure for Efficient Numerical Computation. Comput. Sci. Eng..

[59] SciPy Scipy.Spatial.CKDTree—SciPy v1.5.4 Reference Guide. https://docs.scipy.org/doc/scipy/reference/generated/scipy.spatial.cKDTree.html.

[60] Hunter J.D. (2007). Matplotlib: A 2D Graphics Environment. Comput. Sci. Eng..

[61] Ramachandran P., Varoquaux G. (2011). Mayavi: 3D Visualization of Scientific Data. Comput. Sci. Eng..

[62] Gaillac R., Pullumbi P., Coudert F.-X. (2016). ELATE: An Open-Source Online Application for Analysis and Visualization of Elastic Tensors. J. Phys. Condens. Matter.

[63] Hoogendoorn E., van Golen K. Numpy-Indexed PyPI. https://pypi.org/project/numpy-indexed/.

[64] Lam S.K., Pitrou A., Seibert S. Numba: A LLVM-Based Python JIT Compiler. Proceedings of the Second Workshop on the LLVM Compiler Infrastructure in HPC.

