# Supplementary material for: Effective Mechanical Properties of Periodic Cellular Solids with Generic Bravais Lattice Symmetry via Asymptotic Homogenization
Source: Materials (Basel). 2023 Dec 8;16(24):7562. doi: 10.3390/ma16247562 (PMC10744720; doi:10.3390/ma16247562)
Supplement: Supplementary file 1 [file materials-16-07562-s001.zip › materials-2705381-supplementary.pdf]

Appendix A – 2D Lattice

In this Appendix, the elastic properties of several chosen 2D lattice are plotted. The Homogenized elastic tensor of the following lattices shown in Figure A1 are tabulated in Table A1. The tabulated tensors are based on voxels that are generated as a single layer with an additional periodicity added out of plane. Considering this periodicity means that the single layer is equivalent to a fully tessellated geometry that has infinite depth in the out of plane direction. The tabulated tensors are obtained by fitting a third order polynomial for each of the tensor entries where the volume fraction varies from 0.1 to 0.9.

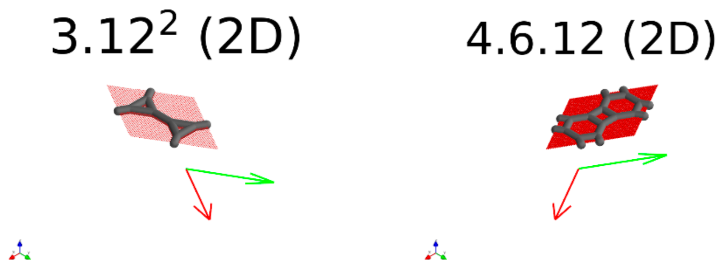

Figure A1: (3.12^2), and (4.6.12) cells with non-orthogonal periodic basis. The representative volume element of the unit cell is shown as red voxels.

The discretized geometry shown in Figure A1 is shown for multiple volume fraction in Figure A2. In this Figure, it can be seen how the geometry of the lattice changes as the volume fraction increases. This sort of filling is one of the main causes of the difference between the theories that use the Euler-Bernoulli beam formulations of homogenization, because it cannot predict the interactions of the geometry within the lattice. The stiffness tensor for the selected lattices in Figure A2 are tabulated in Table A1.

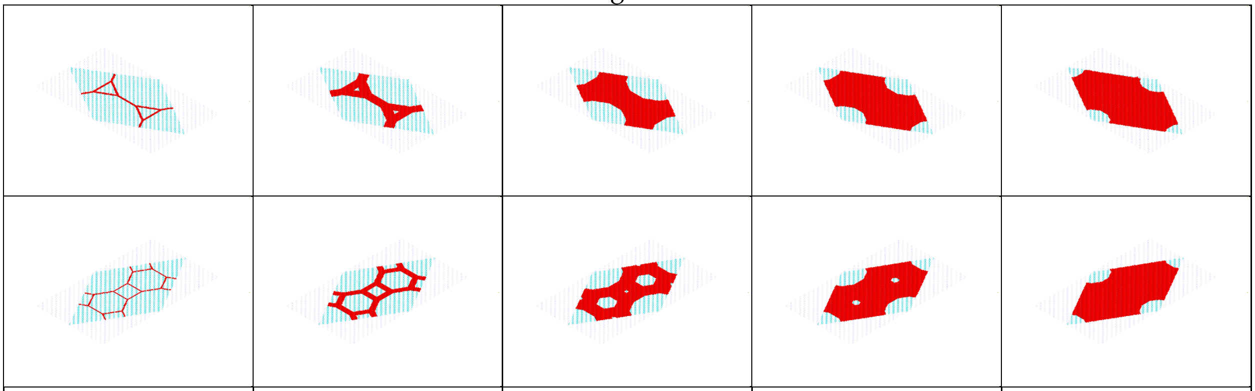

$\rho \cong 0.05$	$\rho \cong 0.28$	$\rho \cong 0.56$	$\rho \cong 0.75$	$\rho \cong 0.86$

Figure A2: Evolution of selected 2D latices as the volume fraction increases

Table A1: Homogenized elastic tensor coefficients (based on Equation **Error! Reference source not found.**) and correlation coefficient for the polynomial fit for chosen 2D lattices

		C_{11}	C_{22}	C_{33}	C_{44}	C_{55}	C_{66}	C_{12}	C_{13}	C_{23}
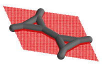 3.12² (2D)	ρ^3	0.776	0.835	0.291	-0.017	0.118	0.098	0.811	0.476	0.494
	ρ^2	0.228	0.182	-0.082	0.443	0.084	0.103	-0.659	-0.129	-0.143
	ρ	0.294	0.266	1.134	-0.052	0.183	0.186	0.386	0.204	0.196
	R	1.000	1.000	1.000	1.000	1.000	1.000	0.998	0.999	0.999
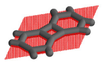 4.6.12 (2D)	ρ^3	0.670	0.631	0.281	-0.119	0.088	0.122	0.909	0.474	0.462
	ρ^2	0.336	0.380	-0.072	0.540	0.123	0.080	-0.757	-0.126	-0.113
	ρ	0.228	0.223	1.106	-0.073	0.165	0.177	0.382	0.183	0.181
	R	1.000	1.000	1.000	0.999	1.000	1.000	0.998	1.000	1.000

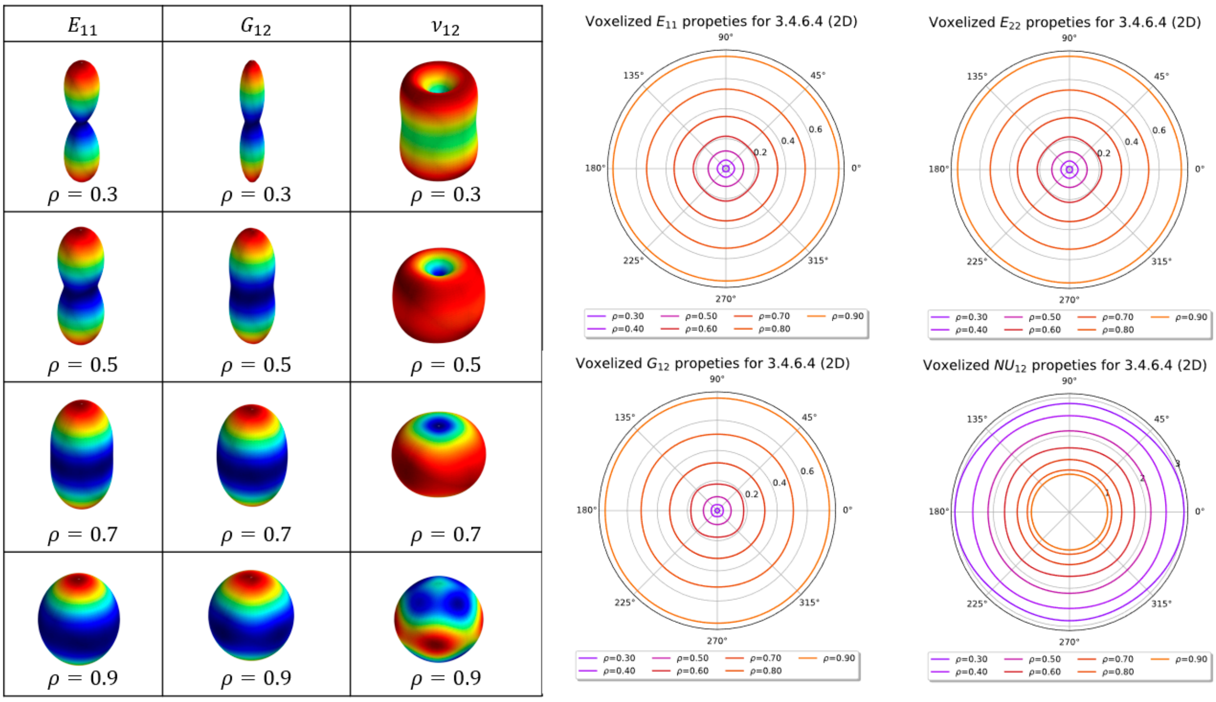

Figure A3: 2D and 3D anisotropy plots for 3.4.6.4 Lattice.

Applying the tensor rotations discussed for the elastic tensors, the 3D anisotropy plots can be obtained even for 2D lattices, with an assumption of infinite depth in the out of plane direction imposed by the periodicity boundary conditions. Due to the infinite depth assumption, the asymptotically homogenized stiffness along the out of plane direction has the most stiffness for 2D lattice and varies linearly with the relative density of the lattice. The variation of the 2D and the 3D anisotropy plots for some elastic properties of 3.4.6.4 lattice are shown in Figure A3. The 3D anisotropy plots are not to scale, and the individual rings in the 2D anisotropy plots corresponds to a horizontal planar intersection of the 3D anisotropy plots.

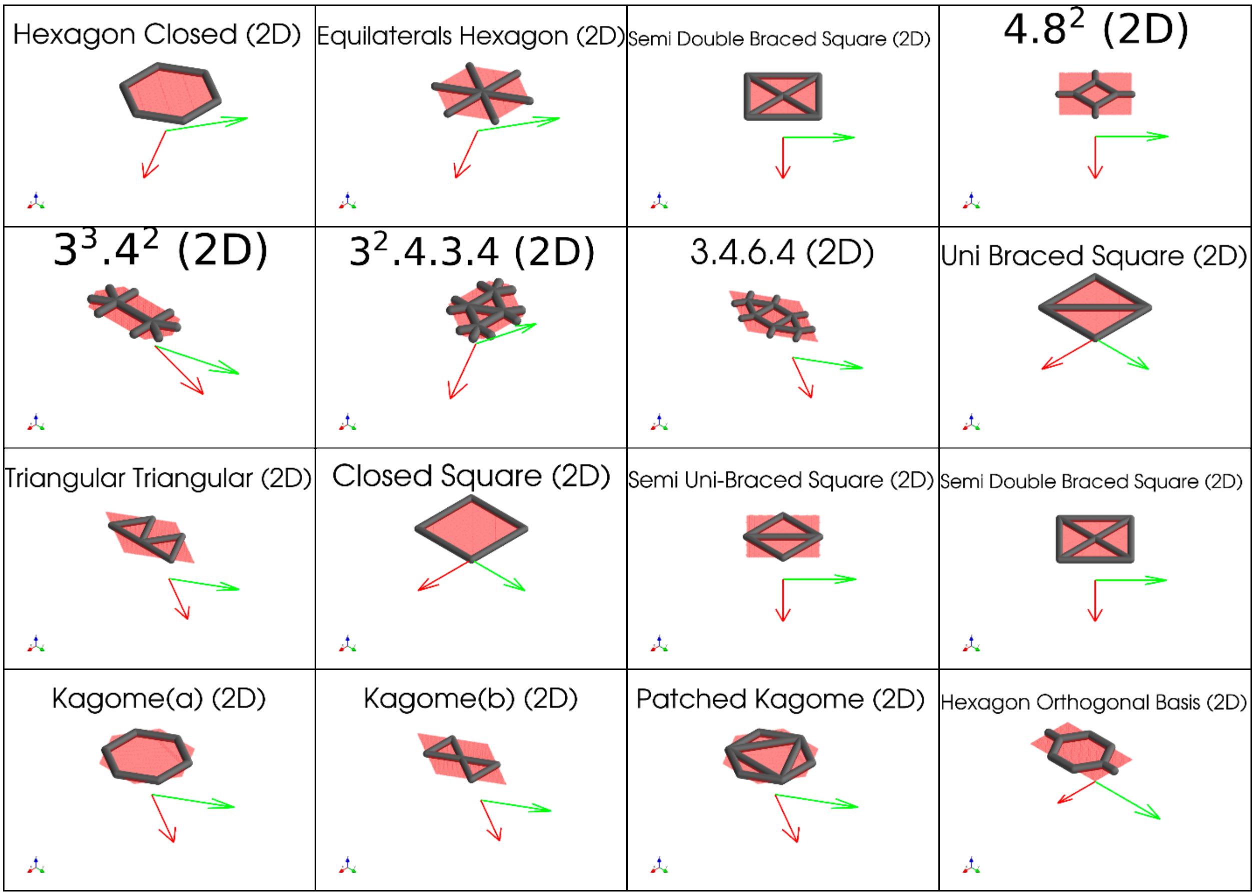

Figure A4: List of all 2D lattice topologies and its corresponding periodic basis.

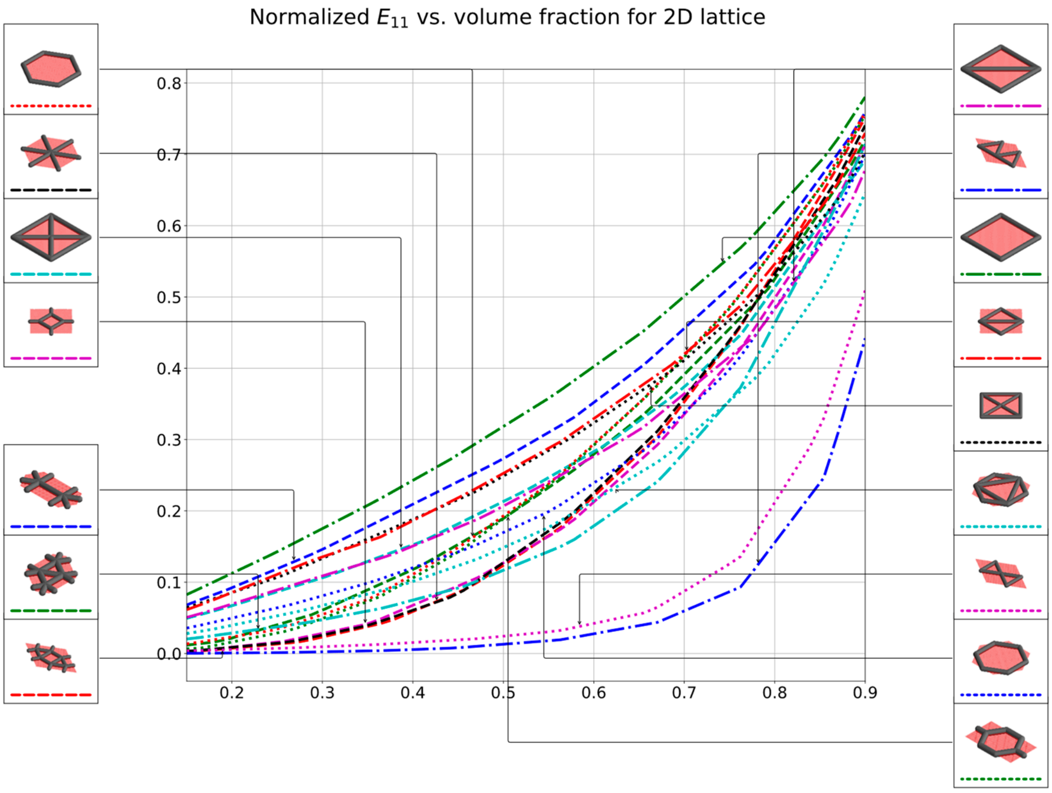

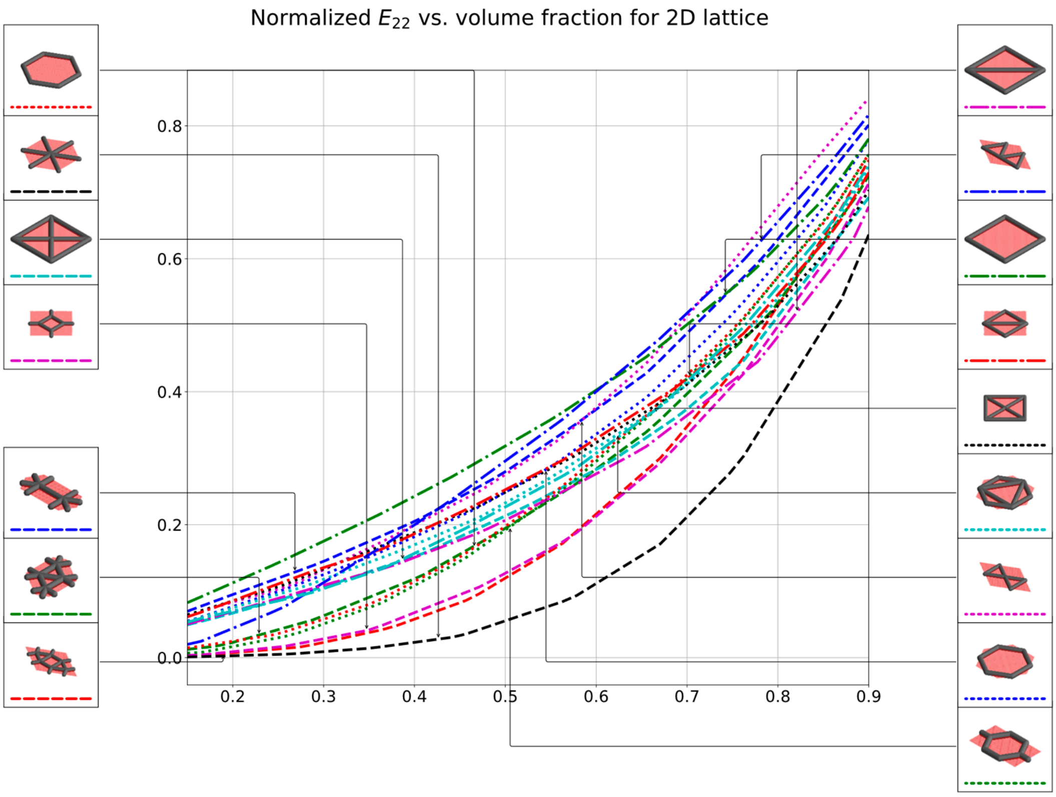
Normalized E_{22} vs. volume fraction for 2D lattice
0.0
0.2
0.4
0.6
0.8
0.2
0.3
0.4
0.5
0.6
0.7
0.8
0.9

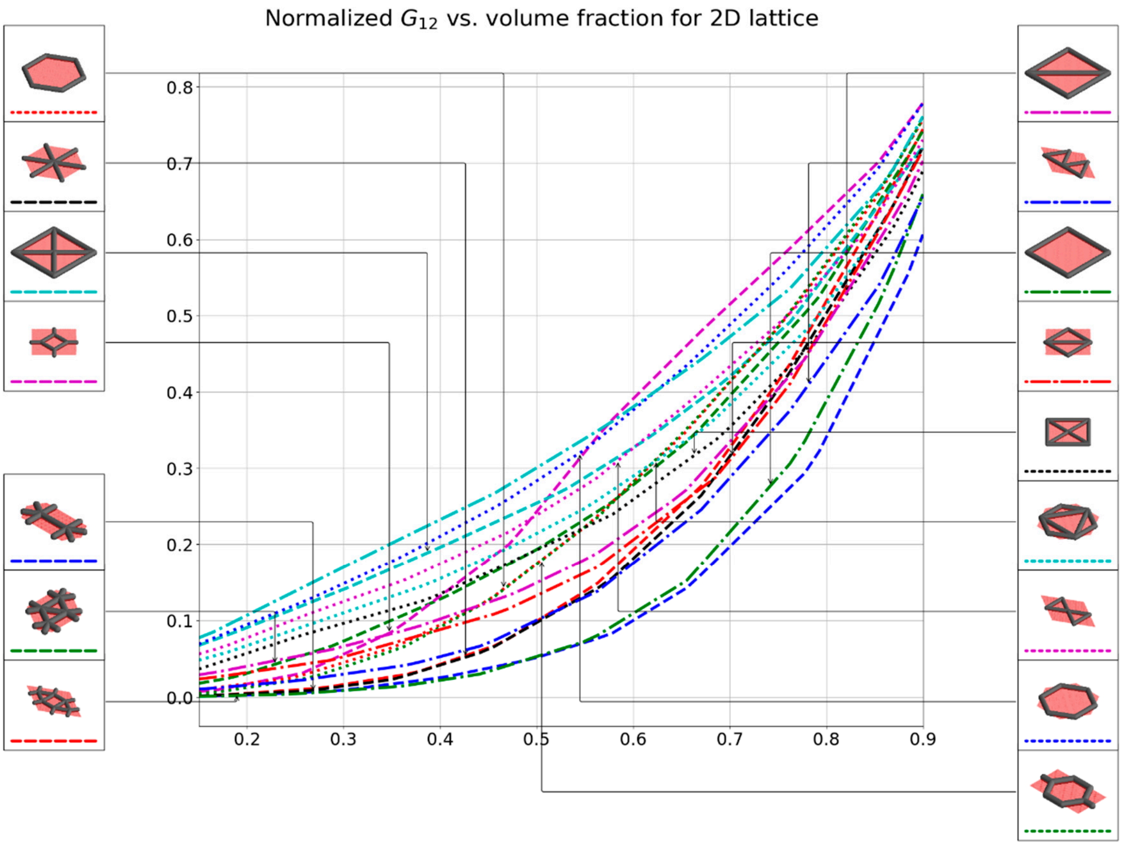
Normalized G_{12} vs. volume fraction for 2D lattice
0.0
0.1
0.2
0.3
0.4
0.5
0.6
0.7
0.8
0.2
0.3
0.4
0.5
0.6
0.7
0.8
0.9

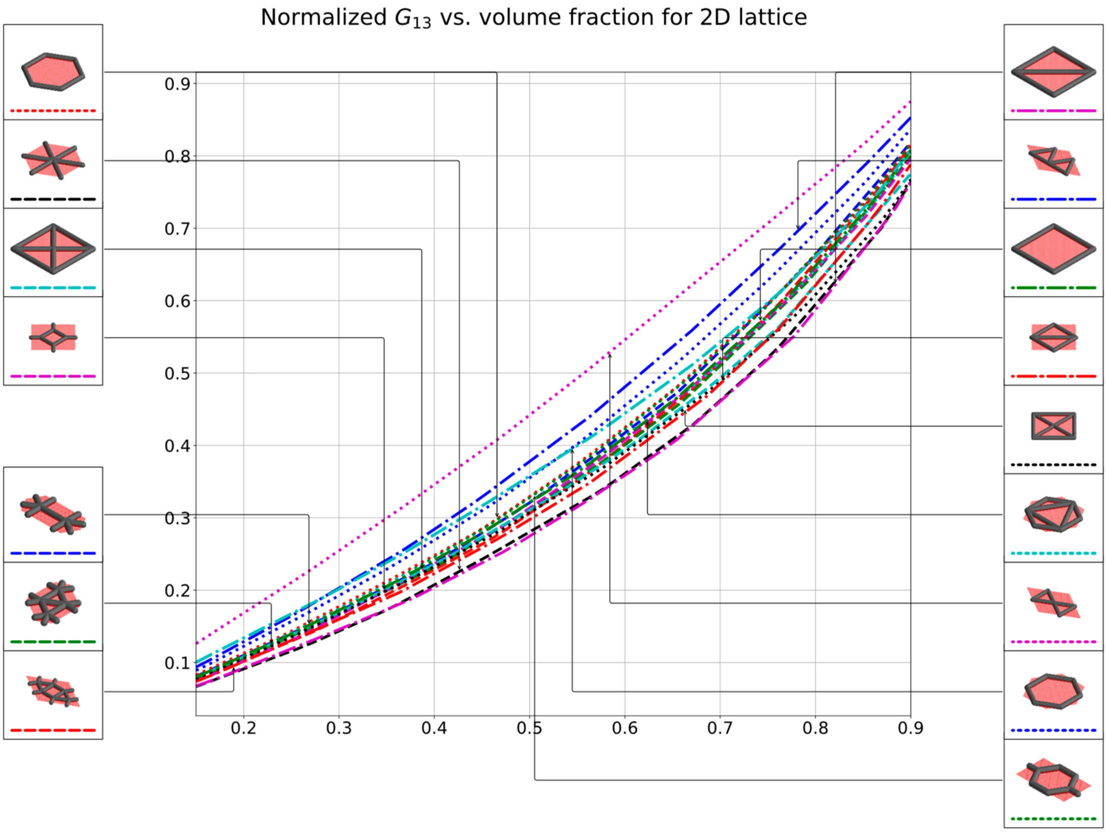
Normalized G_{13} vs. volume fraction for 2D lattice
0.9
0.8
0.7
0.6
0.5
0.4
0.3
0.2
0.1
0.2
0.3
0.4
0.5
0.6
0.7
0.8
0.9

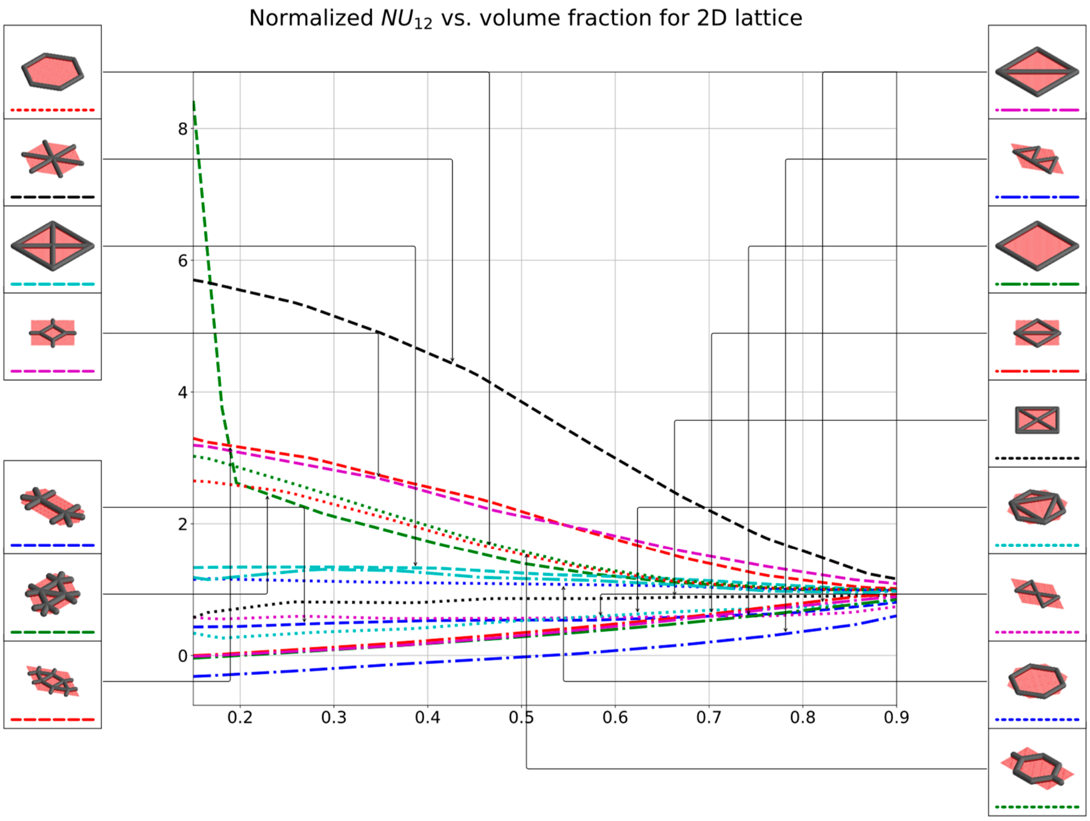
Normalized NU_{12} vs. volume fraction for 2D lattice
8
6
4
2
0
0.2
0.3
0.4
0.5
0.6
0.7
0.8
0.9

Appendix B – 3D Lattice

In this section the homogenized elastic tensor coefficients of chosen 3D lattices are presented in Table B1. The 2D and 3D anisotropy plot of elastic properties for body centered lattice (called X grid) is shown in Figure B1.

Table B1: Homogenized elastic tensor coefficients (based on Equation **Error! Reference source not found.**) and correlation coefficient for the polynomial fit for chosen 3D lattices.

		C_{11}	C_{22}	C_{33}	C_{44}	C_{55}	C_{66}	C_{12}	C_{13}	C_{23}
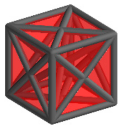	ρ^3	1.452	1.452	1.452	0.335	0.335	0.335	0.768	0.768	0.768
	ρ^2	-0.543	-0.543	-0.543	-0.079	-0.079	-0.079	-0.517	-0.517	-0.517
	ρ	0.369	0.369	0.369	0.125	0.125	0.125	0.232	0.232	0.232
X Cross Grid	R	0.999	0.999	0.999	1.000	1.000	1.000	0.994	0.994	0.994
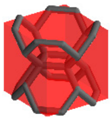	ρ^3	0.628	0.628	0.628	0.046	0.046	0.046	0.740	0.740	0.740
	ρ^2	0.455	0.455	0.455	0.369	0.369	0.369	-0.339	-0.339	-0.339
	ρ	0.244	0.244	0.244	-0.015	-0.015	-0.015	0.177	0.177	0.177
Vintiles (3D)	R	1.000	1.000	1.000	1.000	1.000	1.000	1.000	1.000	1.000
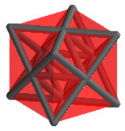	ρ^3	1.612	1.612	1.612	0.192	0.192	0.192	0.801	0.801	0.801
	ρ^2	-0.604	-0.604	-0.604	0.107	0.107	0.107	-0.463	-0.463	-0.463
	ρ	0.376	0.376	0.376	0.101	0.101	0.101	0.222	0.222	0.222
Octet (3D)	R	1.000	1.000	1.000	1.000	1.000	1.000	0.998	0.998	0.998

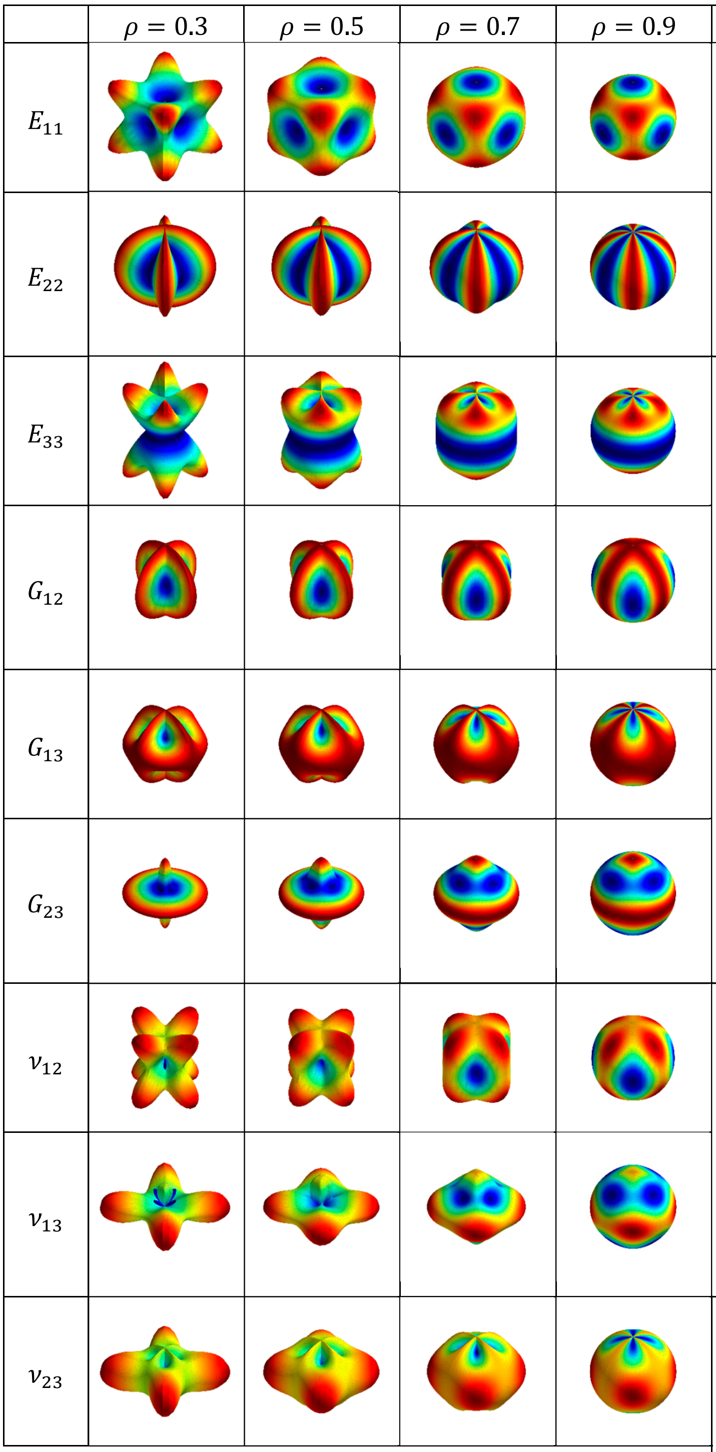

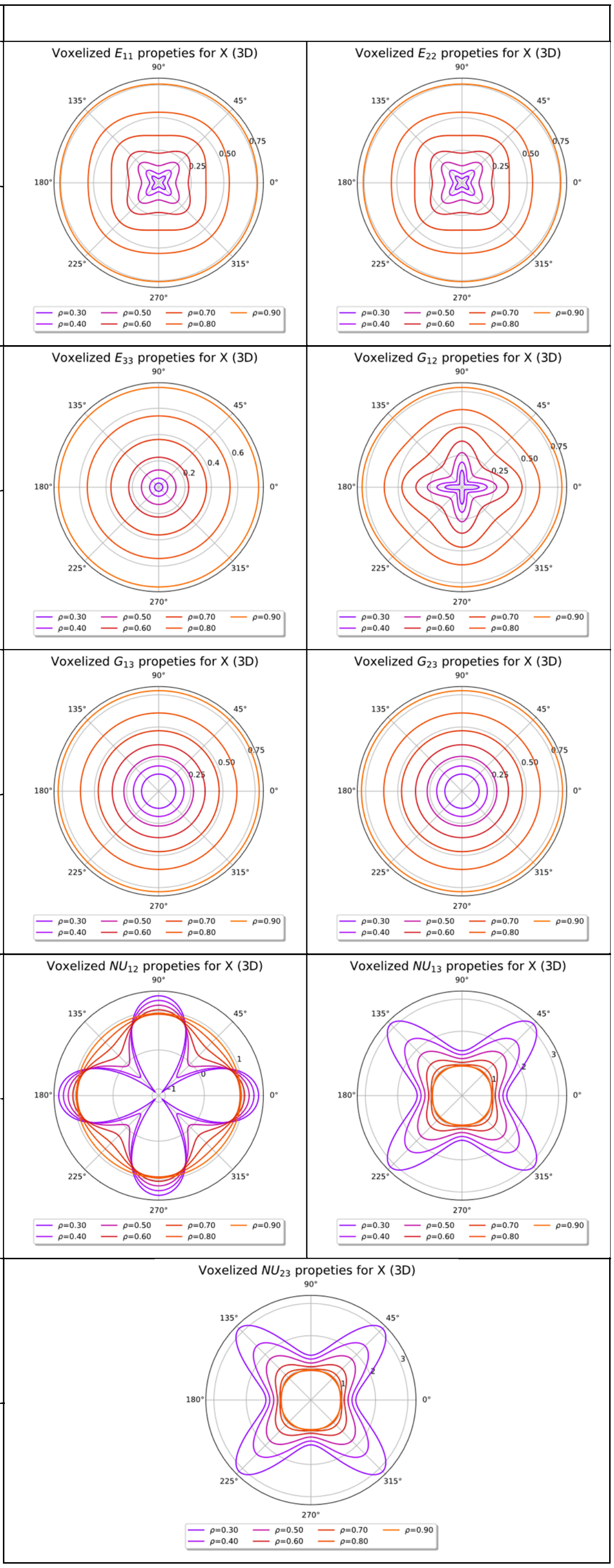

Figure B1: 2D and 3D anisotropy plots for X lattice (3D)

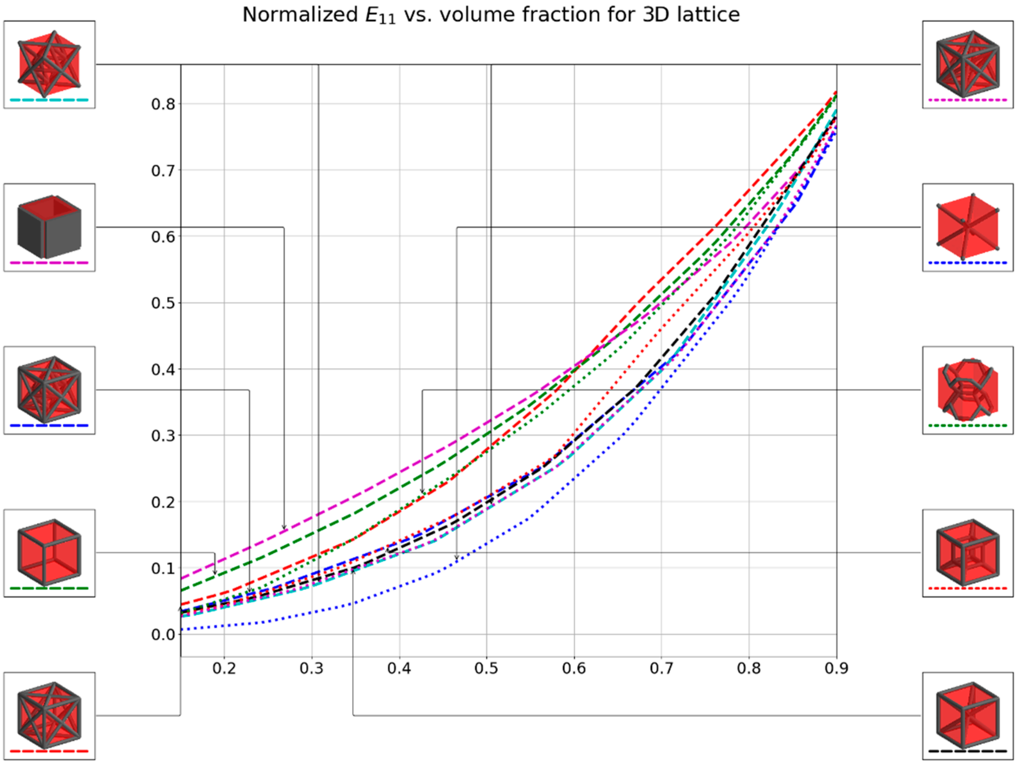

Normalized E_{11} vs. volume fraction for 3D lattice
0.0
0.1
0.2
0.3
0.4
0.5
0.6
0.7
0.8
0.2
0.3
0.4
0.5
0.6
0.7
0.8
0.9

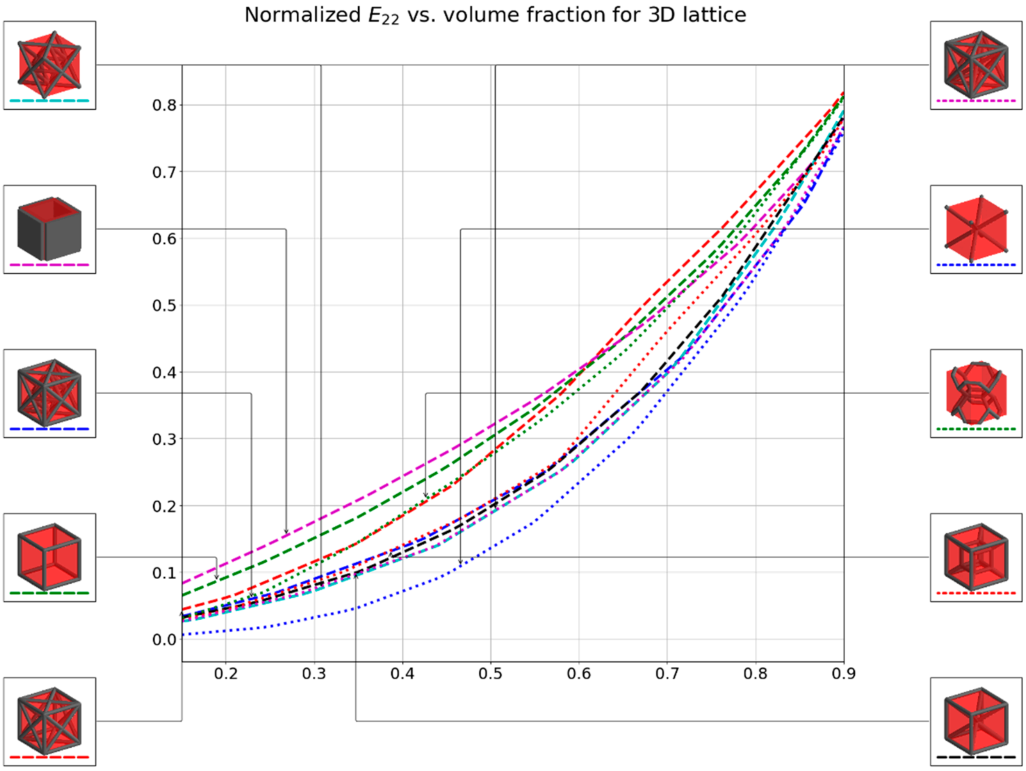

Normalized E_{22} vs. volume fraction for 3D lattice
0.0
0.1
0.2
0.3
0.4
0.5
0.6
0.7
0.8
0.2
0.3
0.4
0.5
0.6
0.7
0.8
0.9

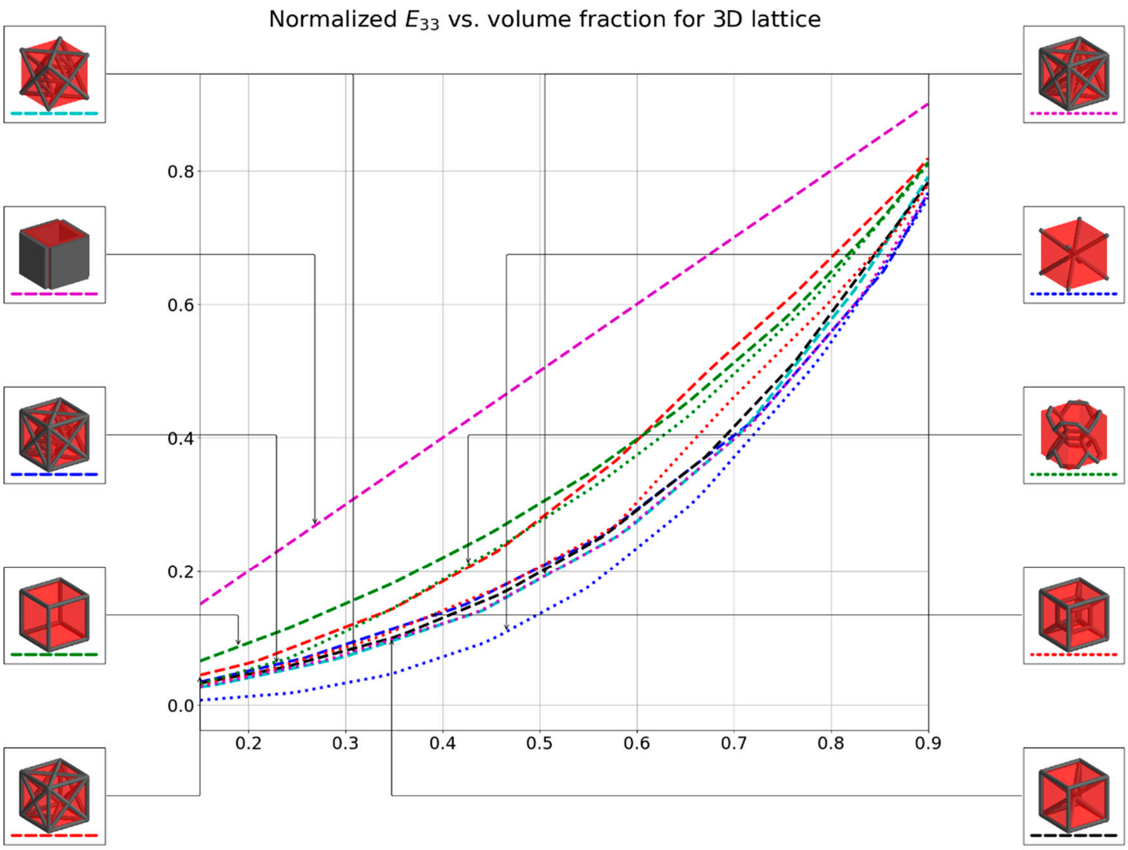
Normalized E_{33} vs. volume fraction for 3D lattice
0.0
0.2
0.4
0.6
0.8
0.2
0.3
0.4
0.5
0.6
0.7
0.8
0.9

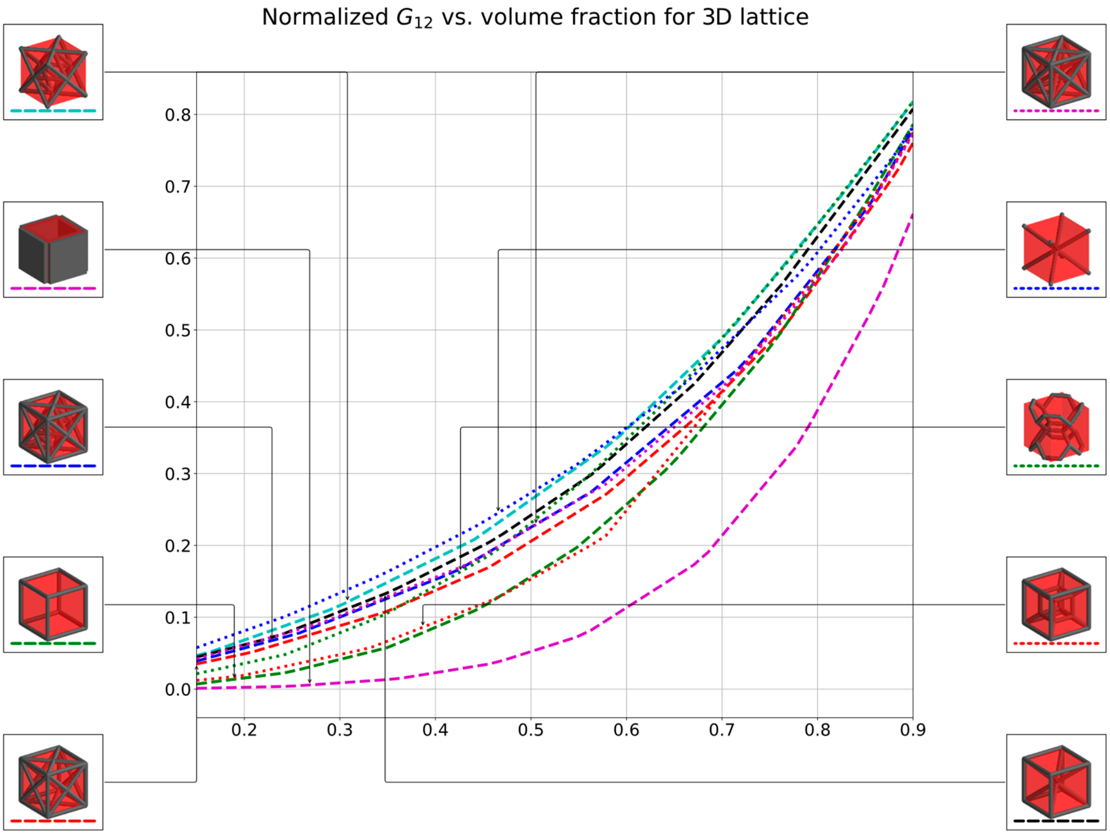
Normalized G_{12} vs. volume fraction for 3D lattice
0.0
0.1
0.2
0.3
0.4
0.5
0.6
0.7
0.8
0.2
0.3
0.4
0.5
0.6
0.7
0.8
0.9

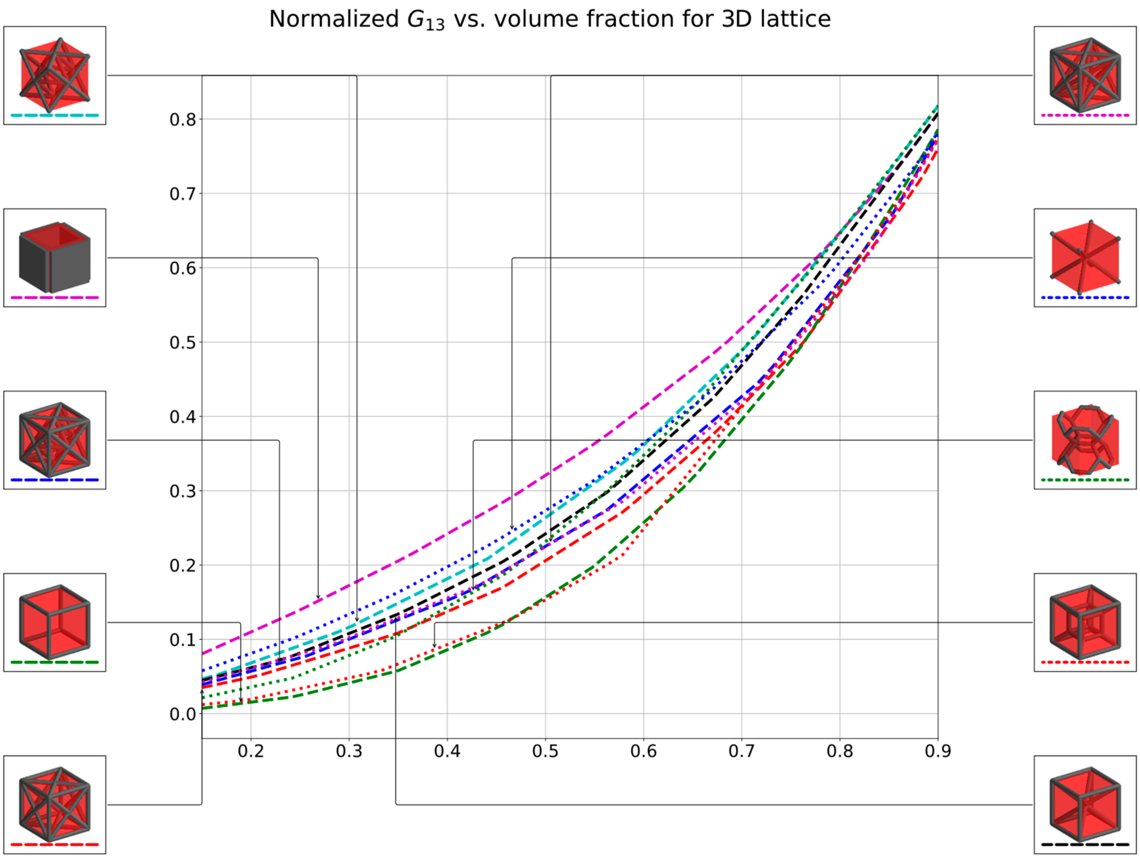
Normalized G_{13} vs. volume fraction for 3D lattice
0.8
0.7
0.6
0.5
0.4
0.3
0.2
0.1
0.0
0.2
0.3
0.4
0.5
0.6
0.7
0.8
0.9

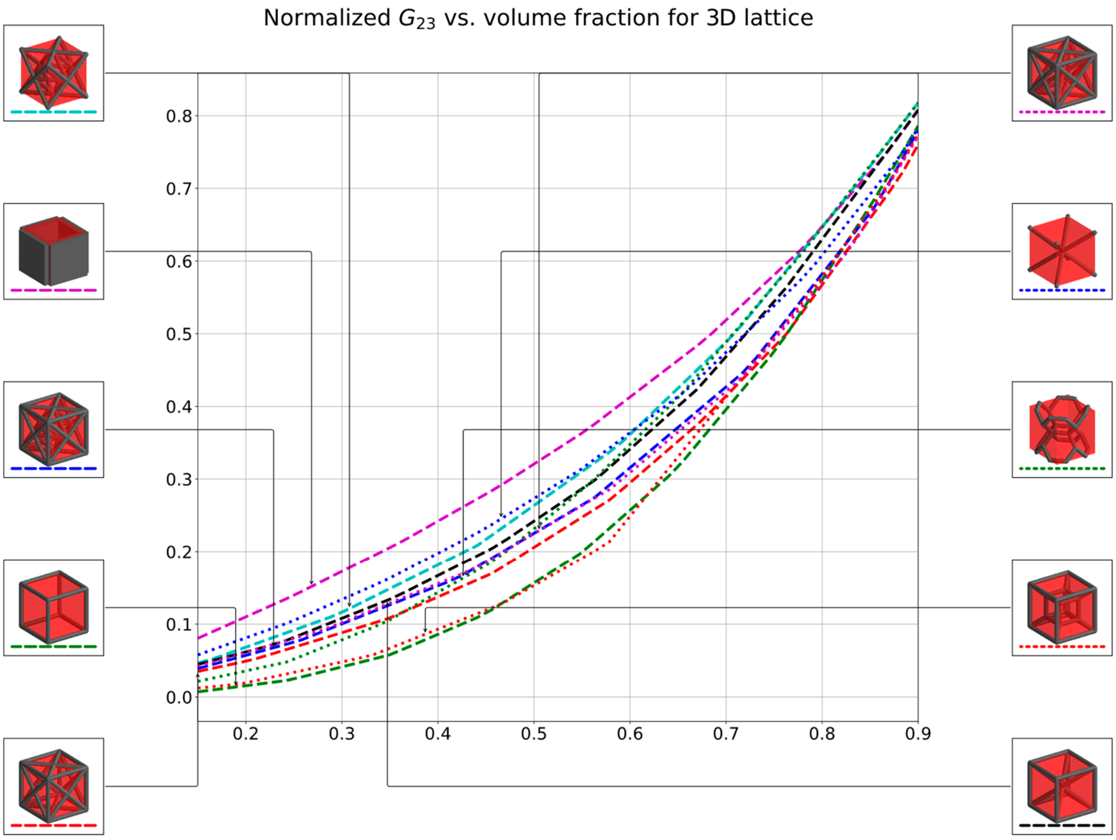
Normalized G_{23} vs. volume fraction for 3D lattice
0.8
0.7
0.6
0.5
0.4
0.3
0.2
0.1
0.0
0.2
0.3
0.4
0.5
0.6
0.7
0.8
0.9

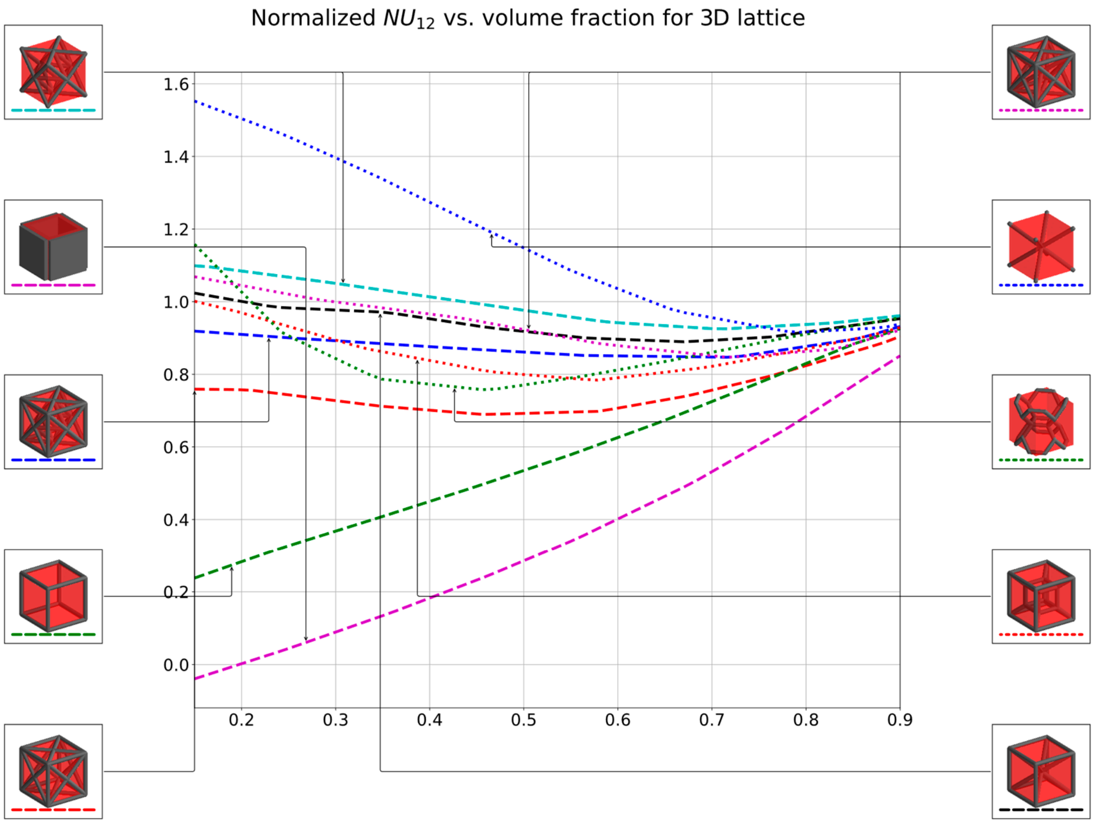
Normalized NU_{12} vs. volume fraction for 3D lattice
1.6
1.4
1.2
1.0
0.8
0.6
0.4
0.2
0.0
0.2
0.3
0.4
0.5
0.6
0.7
0.8
0.9

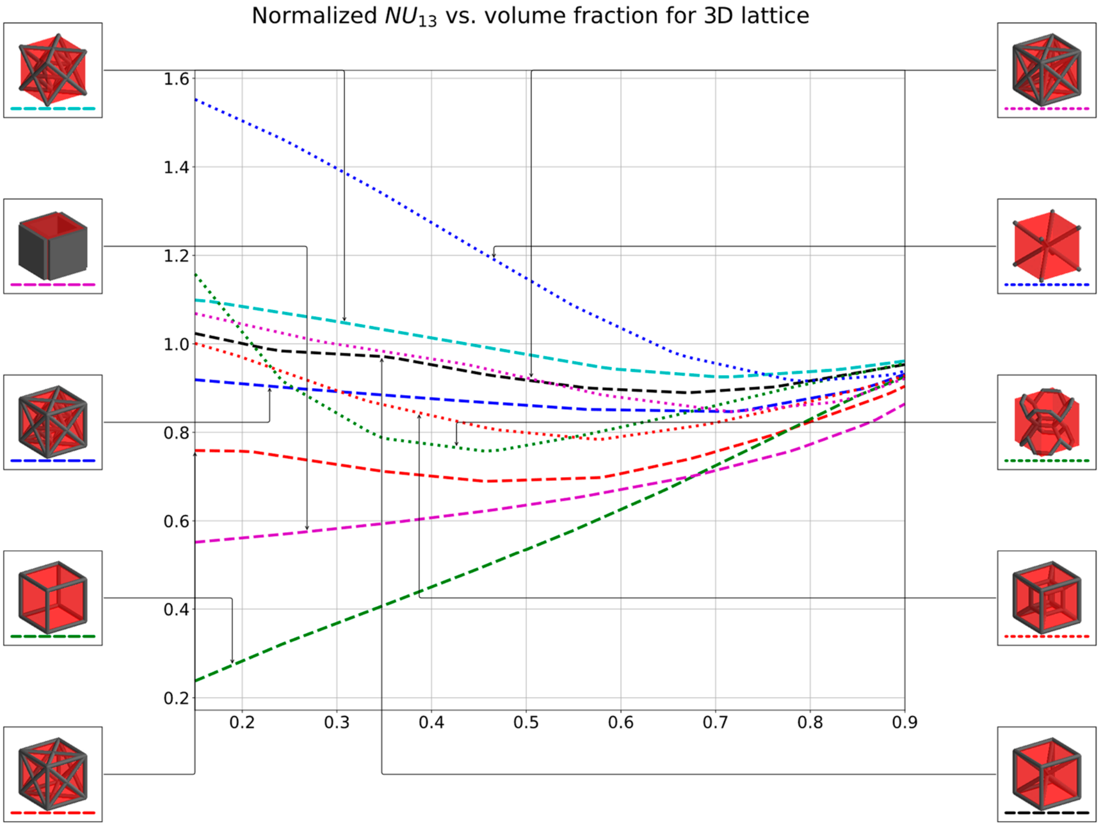
Normalized NU_{13} vs. volume fraction for 3D lattice
1.6
1.4
1.2
1.0
0.8
0.6
0.4
0.2
0.2
0.3
0.4
0.5
0.6
0.7
0.8
0.9

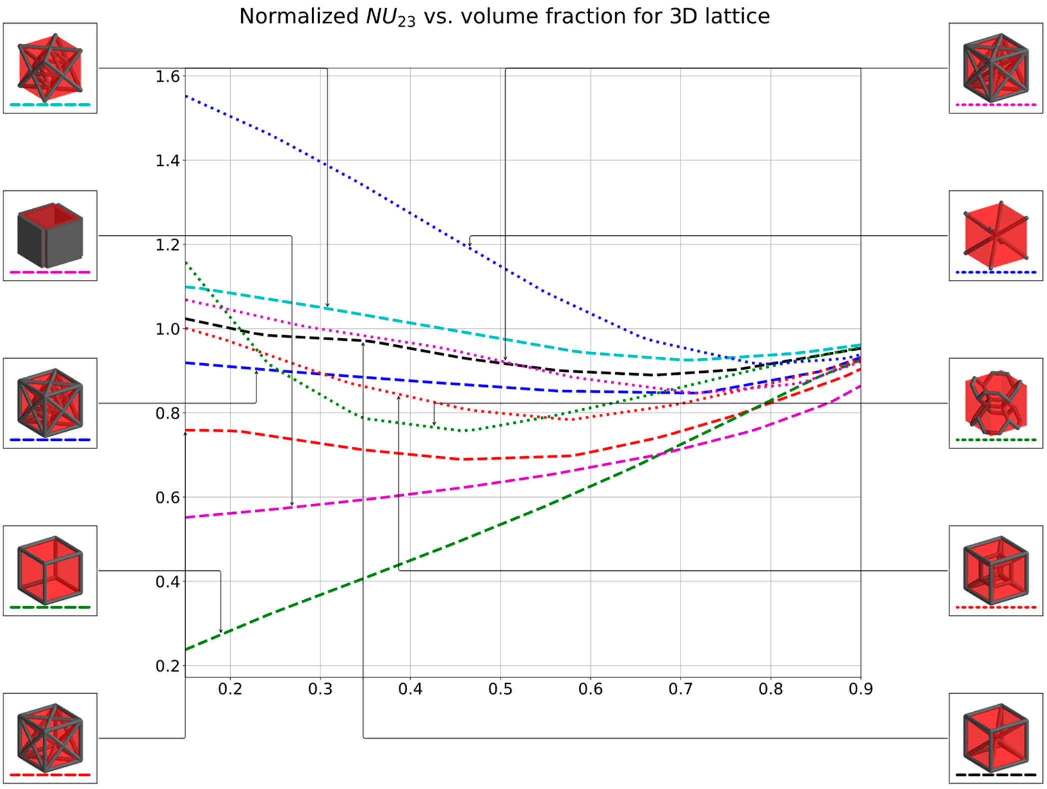

Normalized NU_{23} vs. volume fraction for 3D lattice
1.6
1.4
1.2
1.0
0.8
0.6
0.4
0.2
0.2
0.3
0.4
0.5
0.6
0.7
0.8
0.9

Appendix C – 3D Sandwich Lattice

In this Appendix, the elastic properties of several chosen 3D Sandwich lattice are plotted. For the sandwich panels, a periodicity in the z direction is assumed, thus the elastic properties of the 3D sandwich panels in this section are assumed to be stacked.

The variation of the 3D lattices with sandwich panels are plotted. For the sandwich panels, a periodicity in the z direction (sandwich plate normal) is assumed, thus the elastic properties that are plotted in this section are for sandwich panels that are assumed to be stacked. For all the geometries in this section, the thickness of the sandwich panel is held constant at a unit of 0.05 of the cell length. The 2D and 3D anisotropy plot of elastic properties for X lattice.

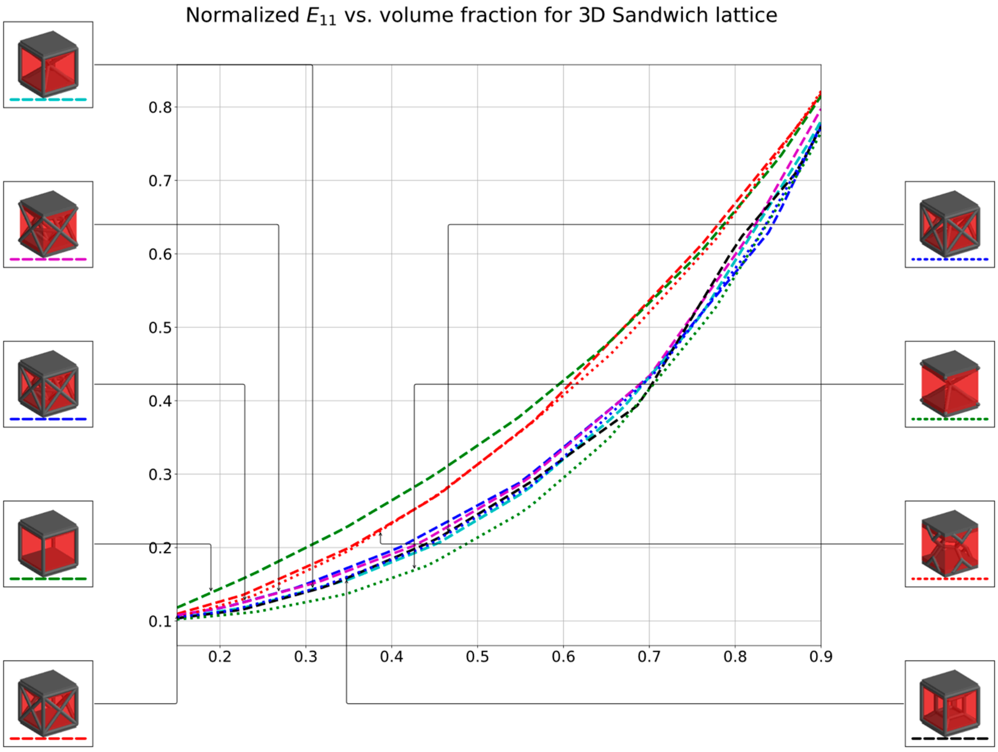

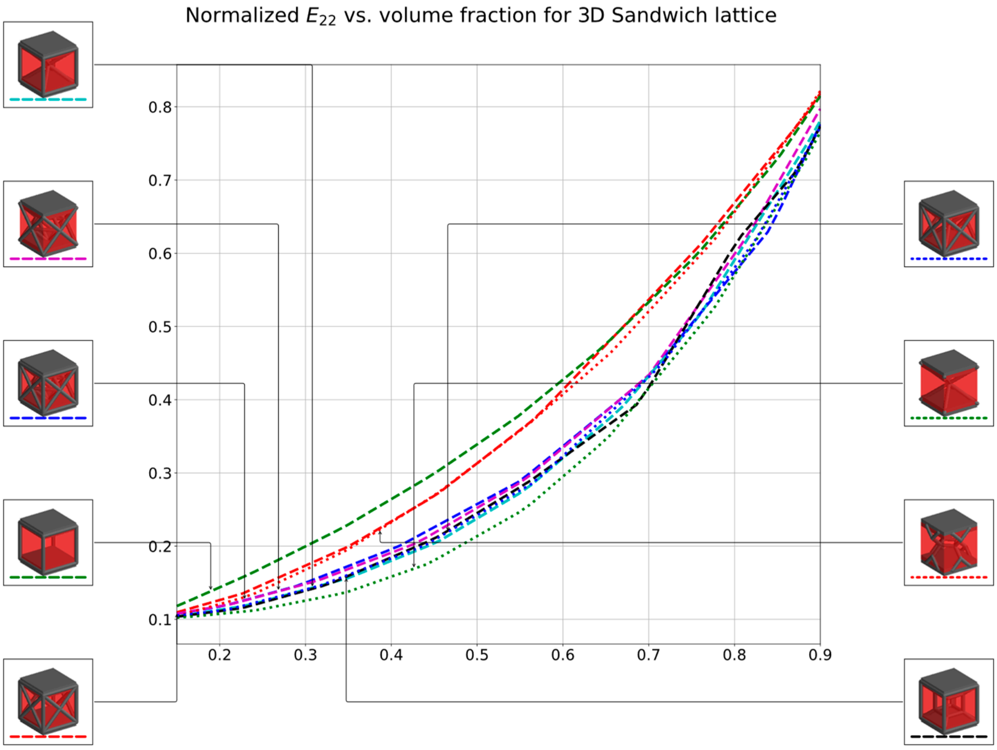
Normalized E_{22} vs. volume fraction for 3D Sandwich lattice
0.8
0.7
0.6
0.5
0.4
0.3
0.2
0.1
0.2
0.3
0.4
0.5
0.6
0.7
0.8
0.9

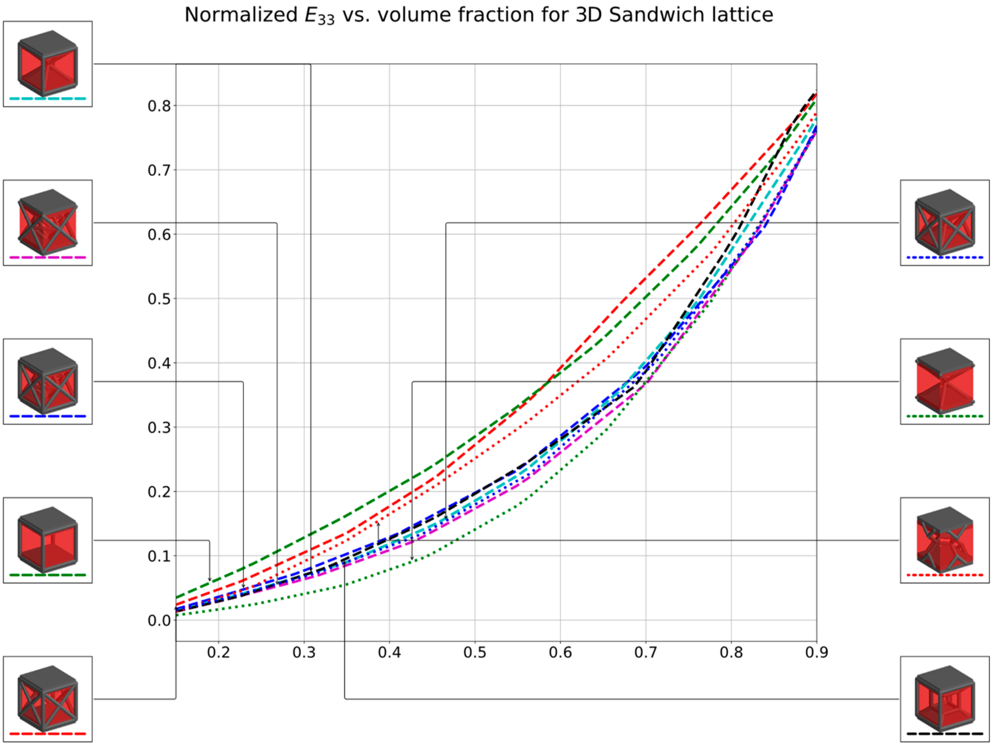
Normalized E_{33} vs. volume fraction for 3D Sandwich lattice
0.8
0.7
0.6
0.5
0.4
0.3
0.2
0.1
0.0
0.2
0.3
0.4
0.5
0.6
0.7
0.8
0.9

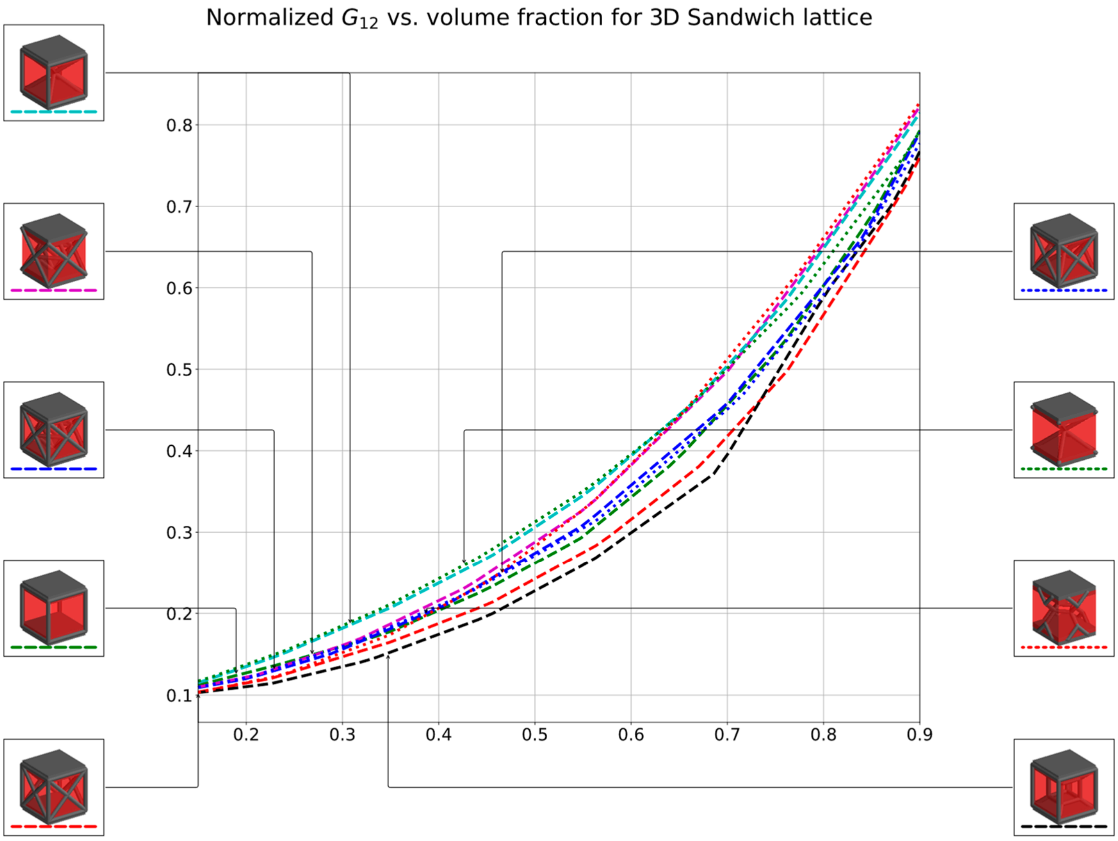
Normalized G_{12} vs. volume fraction for 3D Sandwich lattice
0.8
0.7
0.6
0.5
0.4
0.3
0.2
0.1
0.2
0.3
0.4
0.5
0.6
0.7
0.8
0.9

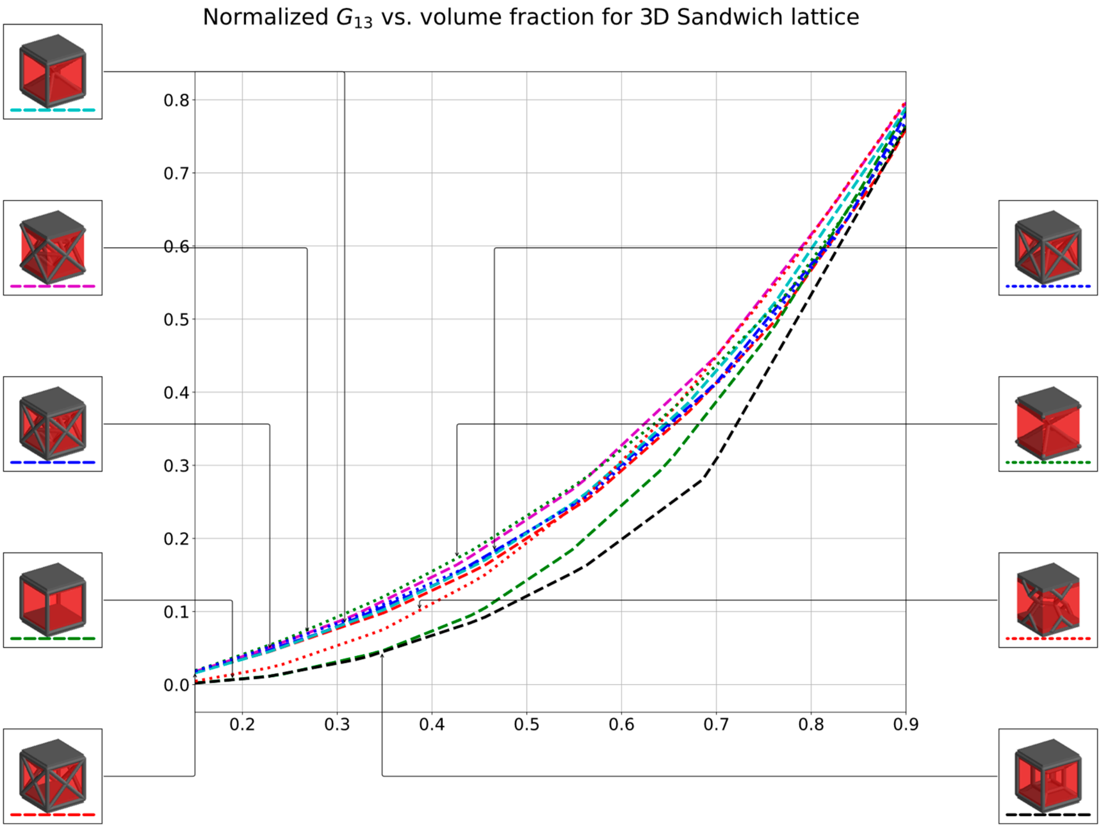
Normalized G_{13} vs. volume fraction for 3D Sandwich lattice
0.8
0.7
0.6
0.5
0.4
0.3
0.2
0.1
0.0
0.2
0.3
0.4
0.5
0.6
0.7
0.8
0.9

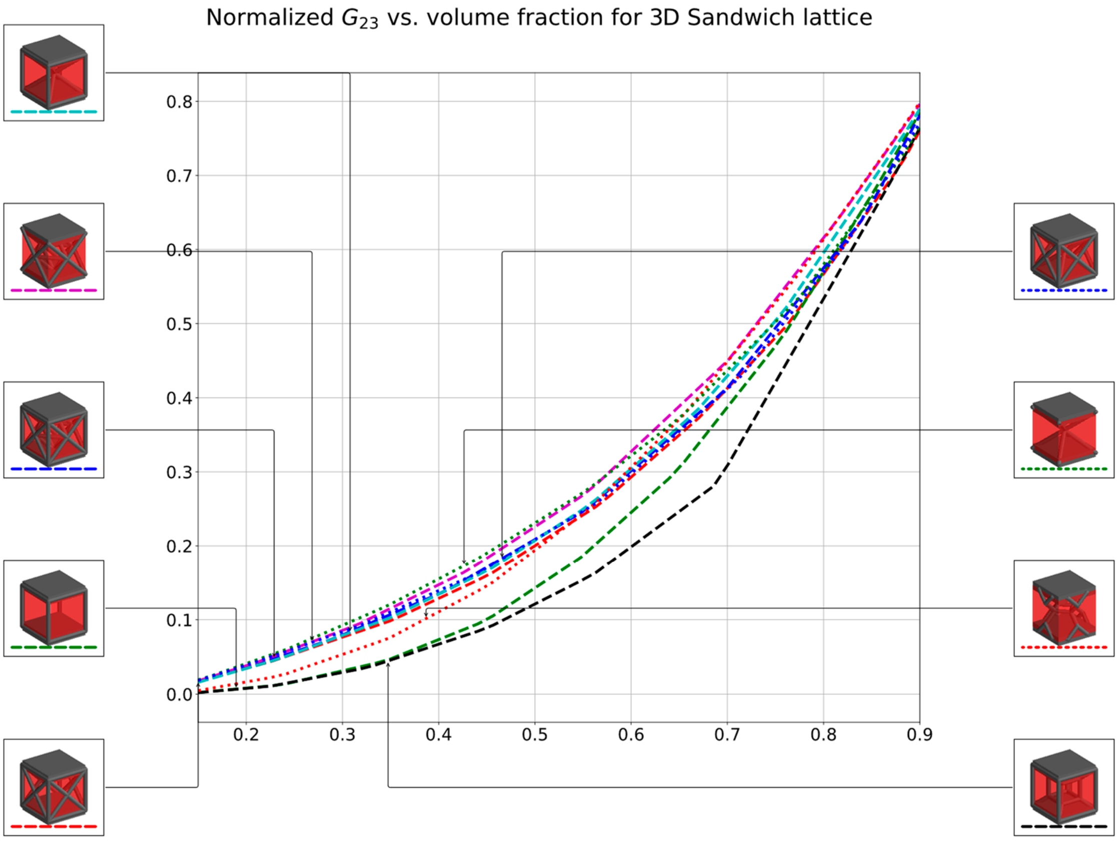

Normalized G_{23} vs. volume fraction for 3D Sandwich lattice
0.8
0.7
0.6
0.5
0.4
0.3
0.2
0.1
0.0
0.2
0.3
0.4
0.5
0.6
0.7
0.8
0.9

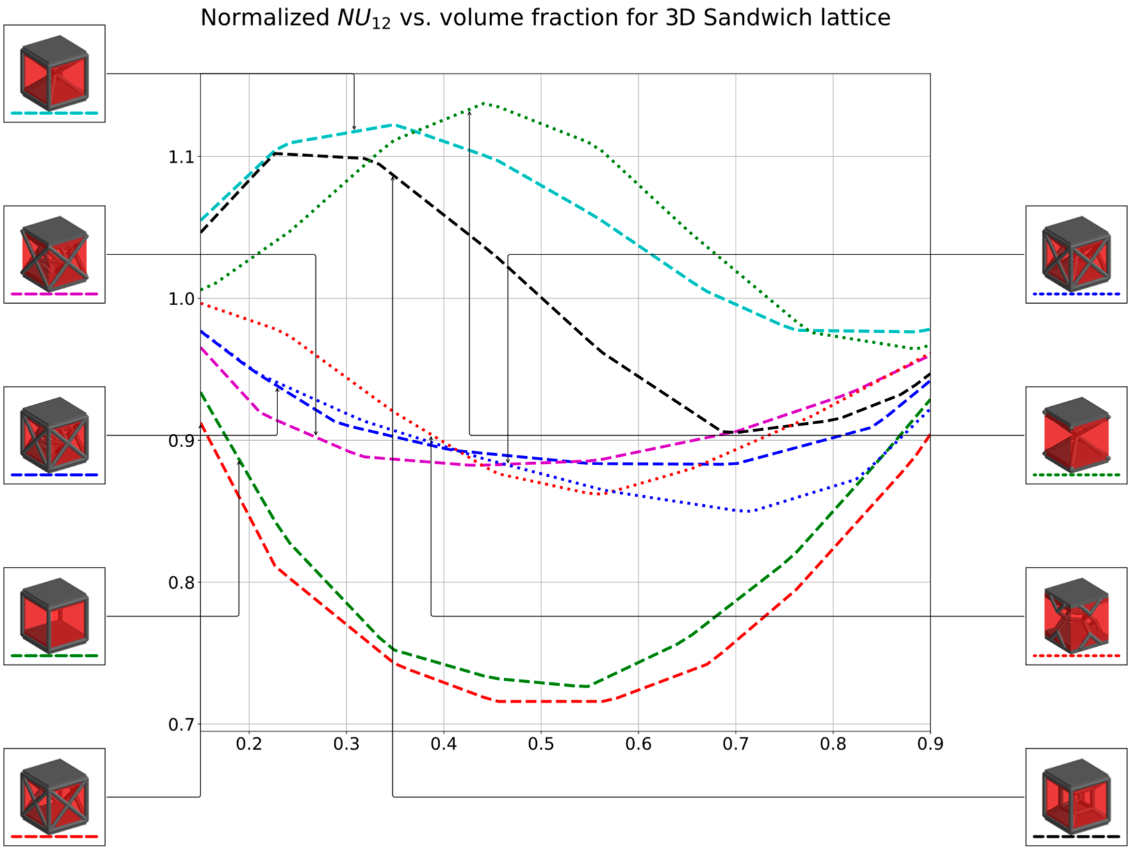

Normalized NU_{12} vs. volume fraction for 3D Sandwich lattice
1.1
1.0
0.9
0.8
0.7
0.2
0.3
0.4
0.5
0.6
0.7
0.8
0.9

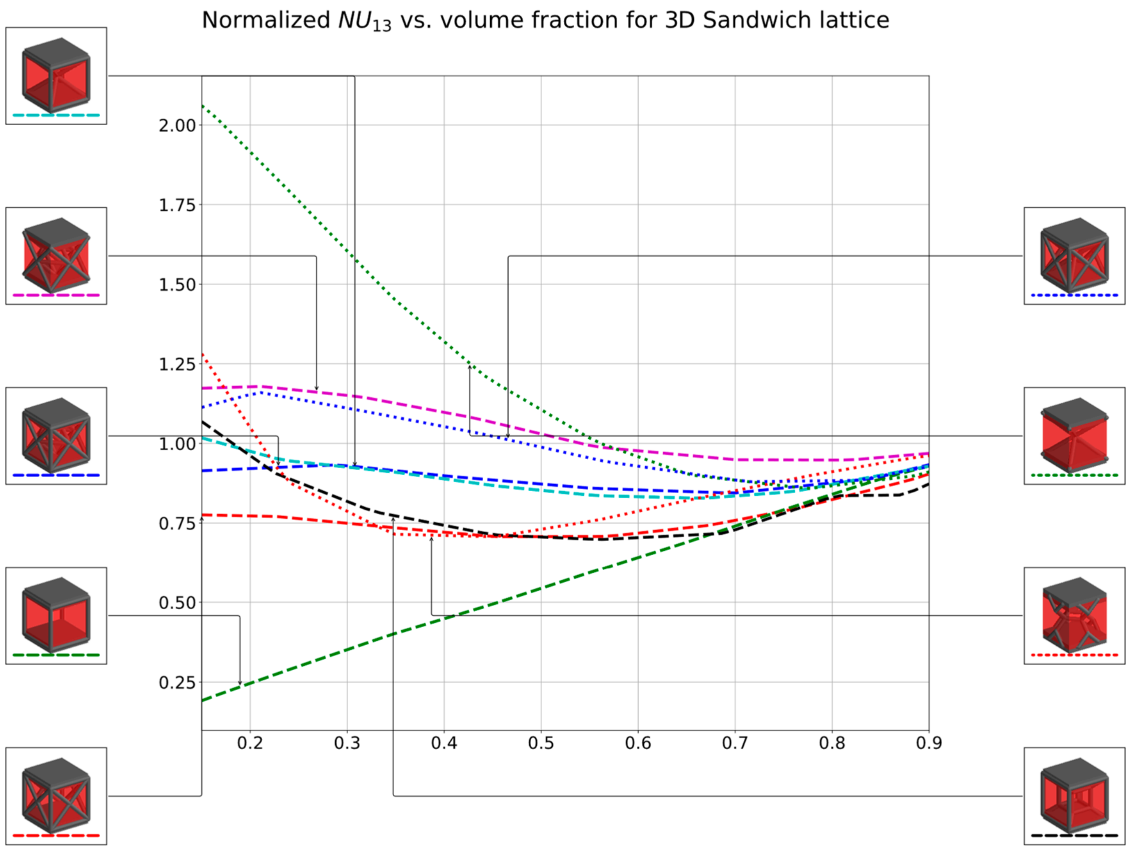
Normalized NU_{13} vs. volume fraction for 3D Sandwich lattice
2.00
1.75
1.50
1.25
1.00
0.75
0.50
0.25
0.2
0.3
0.4
0.5
0.6
0.7
0.8
0.9

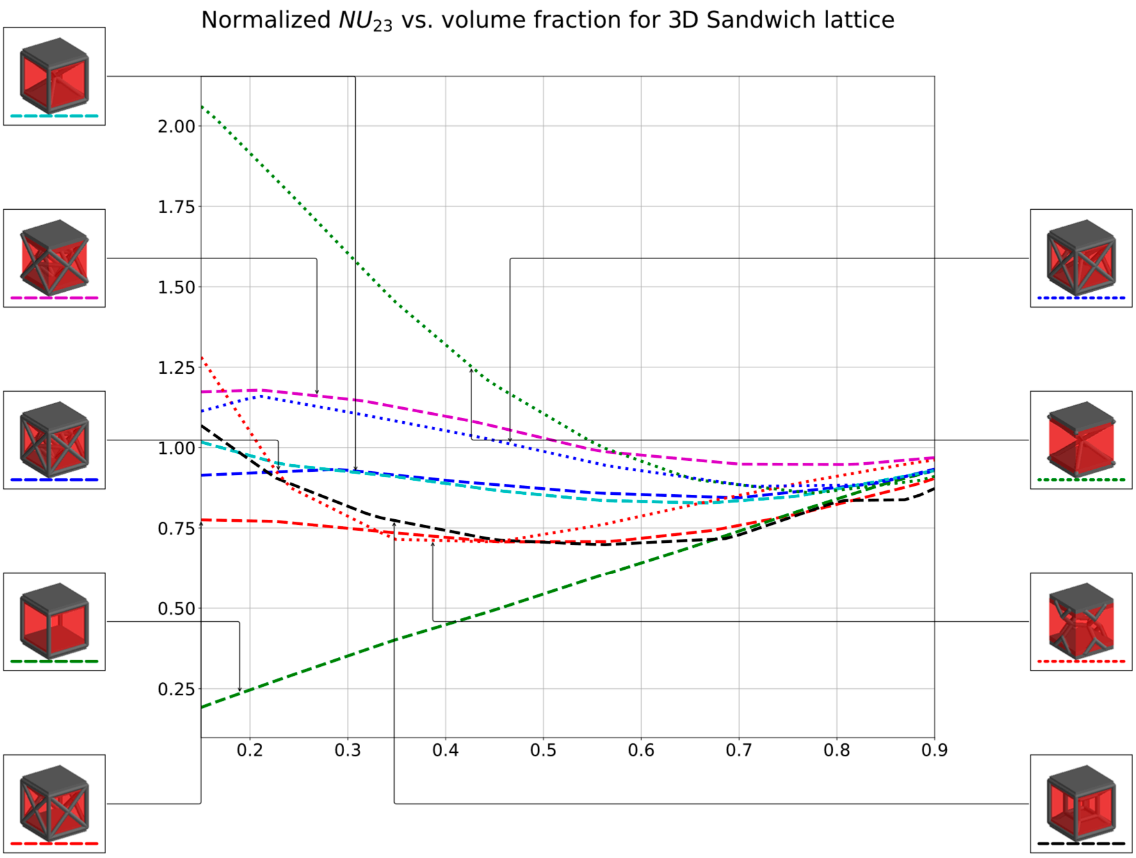
Normalized NU_{23} vs. volume fraction for 3D Sandwich lattice
2.00
1.75
1.50
1.25
1.00
0.75
0.50
0.25
0.2
0.3
0.4
0.5
0.6
0.7
0.8
0.9
